# Cell death-based treatment of ovarian cancer

**DOI:** 10.1038/s41419-026-09133-x

**Published:** 2026-08-03

**Authors:** Aygun R. Mamedova, Gelina S. Kopeina, Boris Zhivotovsky, Larisa V. Smolyaninova

**Affiliations:** 1 Engelhardt Institute of Molecular Biology, Russian Academy of Sciences, Moscow, Russia; 2 Faculty of Medicine, MV Lomonosov Moscow State University, Moscow, Russia; 3 Institute of Environmental Medicine, Karolinska Institute, Stockholm, Sweden

**Keywords:** Cell death, Ovarian cancer

## Abstract

Ovarian cancer (OC) is the seventh most prevalent type of cancer in women and one of the most lethal gynecological malignancies, due to the poor prognosis (with a 5-year survival rate of about 50%), the lack of specific clinical symptoms, the absence of effective screening, and the fact that the tumor grows without obvious signs. These factors complicate early detection, when clinical intervention is most effective, leading to late diagnosis. Standard treatment for OC includes cytoreductive surgery followed by platinum-based chemotherapy (PBC) with carboplatin and paclitaxel or their analogs. Conversely, despite the initial positive response to chemotherapy, many patients experience disease recurrence and develop chemotherapy resistance, which makes long-term treatment challenging and often results in fatal outcomes. Here, we focus on therapeutic strategies that overcome this resistance by precisely targeting pathways of programmed cell death (PCD), thereby opening new avenues for treatment. The potential of using PCD in OC treatment is being considered, focusing on key regulatory mechanisms: triggering intrinsic and/or extrinsic apoptosis; the complex role of autophagy in both promoting survival and inducing cell death; the emerging relevance of ferroptosis activation through inhibition of GPX4 or blockade of Xc- system; and the potential of using necroptosis and pyroptosis as immunogenic PCD modalities that can activate an immune response against the tumor. The use of these different PCD pathways offers opportunities to sensitize resistant tumors and improve patient outcomes.

## Facts

- Ovarian cancer is one of the most lethal of female cancers.
- Ovarian cancer is frequently diagnosed late, when the treatment is less effective.
- Current treatments still often fail due to the high rate of chemotherapy resistance and the low susceptibility.

## Open questions

- How do cancer cells from different OC subtypes acquire resistance to conventional and non-conventional therapies?
- Can we combine therapies that target various types of cell death to overcome resistance for successful treatment of patients with different OC subtypes?
- Can spatial proteo-transcriptomic profiling of patients for selecting the most effective targets become a successful tool for the treatment of different OC subtypes?

## Introduction

The ovary is the primary reproductive organ (gonad) in females. It fulfills two main physiological functions: (i) maturation and release of female gametes in a periodic manner and (ii) secretion of female sex hormones (mainly estrogen and progesterone) [1]. The ovary undergoes continuous structural and functional changes during the menstrual cycle [2]. Unfortunately, with age, the risk of ovarian cancer (OC) increases. Most cases of OC develop after menopause. It is also more common in women who have ovulated more frequently over their lifetime. The high risk for OC is observed among those who have never had kids, those who began ovulation at a younger age, and those who reach menopause at an older age [3].

**Fig. 1:**
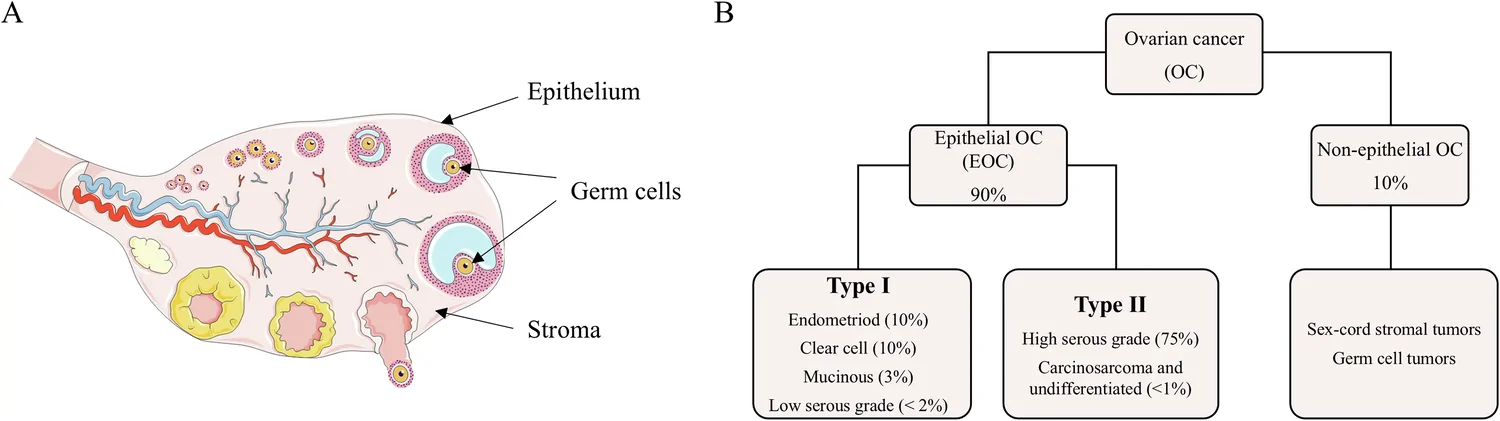
Classification of ovarian cancer. Schematic representation of the main entities of ovarian tumors (**A**) and diagram with different OC subtypes (**B**). Image provided by Servier Medical Art, licensed under CC BY 4.0.

OC is one of the leading gynecological malignancies and the most lethal neoplasm of the female reproductive system, which is diagnosed at an advanced stage (FIGO stage III and IV) with distant metastasis due to a lack of effective screening modalities and the nonspecific symptoms and signs of early-stage disease [4]. OC is a heterogeneous disease with diverse histological subtypes and is classified by the origin of one of the three main ovarian components: epithelium, stroma, and germinal cells (Fig. 1A). Epithelial OC (EOC) is the most common one (90%), and only 10% have a non-epithelial origin (sex-cord stromal tumors and germ cell tumors). Based on origin, prognosis, biology, and clinical and molecular profiles, EOC can be divided into two subtypes (Fig. 1B). Type I includes endometrioid, clear cell (CCC), mucinous, and low-grade serous carcinomas (LGSOC). Type II includes high-grade serous carcinoma (HGSOC), carcinosarcomas, and undifferentiated carcinomas [5, 6]. The main characteristics are given in Table 1.

**Table 1:** Major characteristics of EOC subtypes.

	HGSOC	LGSOC	CCC	Endometrioid	Mucinous
Features	▪ extremely aggressive tumor ▪ large, bilateral, with a solid and cystic appearance ▪ solid areas are often extensively necrotic ▪ solid, papillary, labyrinthine (with slit-like spaces), glandular, cribriform growth patterns, often in combination ▪ rapidly developing cancer	▪ bilateral, solid, and cystic tumors characterized by papillary and nodular growth with calcification ▪ may have areas of benign or borderline morphology ▪ necrosis is rare ▪ slow-growing cancer	▪ combination of tubulocystic, papillary, and solid patterns composed of clear (or oxyphilic) cuboidal and hobnail cells ▪ association with endometriosis	▪ variable glandular, papillary, and solid morphologic patterns ▪ association with endometriosis and adenofibroma ▪ usually low grade ▪ high-grade forms are rare and resemble HGSOC	▪ large, unilateral, multilocular solid and cystic tumors composed of cells with intracytoplasmic mucin ▪ mix of benign, borderline, non-invasive, and invasive components ▪ Presence of gastrointestinal-type epithelium
Immunophenotype	▪ CK7 + , PAX8 + , WT1 + , p16 + , ER + , PR+ ▪ CK20- ▪ Ki67 (↑)	▪ CK7 + , WT1 + , PAX8 + , ER + , PR+ ▪ Ki67 (↓)	▪ CK7 + , PAX8 + , HNF1β + , Napsin A + , AMACR+ ▪ ER-, PR-, WT1-, ARID1A-	▪ CK7 + , ER + , PR + , PAX8+ ▪ WT1-, Napsin A-	▪ CK7 + , CK20 + , CDX2+ ▪ WT1-, Napsin A-, ER-, PR-, Vimentin-
Molecular alterations	*TP53*, *BRCA1/BRCA2* mutations	*KRAS*, *NRAS*, *BRAF*, *USP9X*, *EIF1AX*, *CDKN2A/2B* mutations	*ARID1A*, *PIK3CA*, *TERT*, *KRAS*, *PTEN*, *MMR* gene mutations	*CTNNB1*, *PIK3CA*, *PTEN*, *KRAS*, *ARID1A* mutations	▪ *KRAS*, *TP53*, *CDKN2A* mutations ▪ *ERBB2* amplification
Stage at diagnosis	advanced	early or advanced	early	early	early
Response to PBC	Pt-sensitive (initially, with subsequent resistance)	Pt-resistant	Pt-resistant	Pt-sensitive	Pt-resistant
Prognosis	generally poor	▪ early stages: favorable ▪ advanced stages: poor	▪ early stages: favorable (60-80%) ▪ advanced stages: poor	generally favorable	▪ early stages: favorable (80%) ▪ advanced stages: poor

The main risk factors for the development of OC include genetic predispositions, hormone replacement therapies, higher body mass index, and smoking. The major genetic risk factors are inherited harmful mutations in the tumor suppressor genes *BRCA1* and *BRCA2*, which are responsible for the repair of DNA double-strand breaks (DSBs) and interstrand crosslinks. Modifications to EOC classification and the discovery of mutated oncogenes, which encode activated signaling molecules that drive cellular proliferation and promote tumor growth, have not led to the development of new screening biomarkers or changes in diagnostic strategies in clinical practice, which have remained largely unchanged for decades. The majority of EOС belongs to HGSOC (60%), characterized by bilateral ovarian involvement, aggressive behavior, therapy resistance, and high mortality [7].

Anticancer therapy for newly diagnosed OC includes cytoreductive surgery followed by adjuvant cytotoxic platinum-based chemotherapy (PBC) with carboplatin and paclitaxel or their analogs, causing DNA damage and collapse of microtubules, preventing the cell cycle, and inducing an apoptotic type of programmed cell death (PCD) in rapidly proliferating tumor cells. Although PBC has been successfully used clinically to improve the treatment of OC, most tumors develop resistance to standard treatment, and patients experience relapses. In the last decade, new strategies have emerged in the treatment of OC, such as targeting oncogenic pathways, including DNA repair (poly(ADP-ribose) polymerase (PARP) inhibitors) and tumor angiogenesis; activating the innate immune response; enhancing drug delivery (antibody-drug conjugates); and using immune-promoting agents such as oncolytic viruses and cancer vaccines [8, 9]. However, with the increasing number of newly mentioned targeted agents that are applied in clinical practice, resistance has been found to occur.

Considering that the overall survival (OS) rate remains low, it is necessary to take into account that activation of various cell death modalities (apoptosis, autophagy, necroptosis, ferroptosis, pyroptosis, etc.) may help overcome treatment resistance. Here, we discuss current advances in understanding the molecular pathways of different cell death modalities and related targeted therapies in OC. The cell death mechanisms induced by different treatment strategies and their contributions to therapy resistance will be explored.

## Targeting cell death for OC therapy

PCD is a genetically controlled mechanism of cell death that occurs under physiological conditions and stabilizes the internal environment. They play an essential role during the development and maintenance of multicellular organisms [10]. PCD is broadly categorized into apoptotic and non-apoptotic modalities (autophagy, necroptosis, ferroptosis, pyroptosis, etc.). The balance of PCD pathways is also crucial in modulating tumorigenesis and therapeutic response. Aberrant regulation of these mechanisms contributes to unrestricted cellular proliferation and the development of resistance to conventional anti-cancer therapies [11]. Increasing evidence suggests a strong connection between PCD regulation and the effectiveness of anticancer treatments [12–14].

It is important to mention that at the time of OC diagnosis, nearly 70% of patients already have metastases in the omentum or peritoneum [15]. The dissemination of OC within the peritoneal cavity reflects the interaction between tumor cells and stromal cells. The latter is part of a complex tumor microenvironment (TME) that can be critical for OC development and anti-tumor therapy. TME is considered a dynamic niche that is composed of diverse components, including immune and stromal cells, and vasculature that are embedded within a remodeled extracellular matrix (ECM), which consists of chemokines, inflammatory cytokines, integrins, matrix metalloproteinases, and other secreted molecules that may facilitate tumor cell migration, invasion, and metastasis [16]. At the beginning of OC progression, the interplay among tumor, immune, and stromal cells can lead to the formation of an immunosuppressive niche in specific sites. Still later, such dynamic interactions can foster primary OC and significantly influence the proliferation of cancer cells, angiogenesis, evasion of immune surveillance, apoptosis inhibition, and immune system suppression. The critical role of TME is now increasingly recognized in disease progression, metastasis, and treatment resistance, but the therapeutic approaches targeting the TME have not cured OC; instead, they have shown the potential to control the development of OC. There are numerous cells, such as myeloid-derived suppressor cells, tumor-associated macrophages, regulatory T cells, and cancer-associated fibroblasts, that secrete high numbers of immunosuppressive cytokines within the TME, which is thought to contribute to the lack of success of immunotherapies such as immune checkpoint inhibitor treatment [17]. It is known that immune checkpoint therapy is an important part of maintaining self-tolerance and enhancing an antitumor immune response, but for the improvement of therapeutic response in combination with cytotoxic chemotherapy, it is essential for PCD activation. It has been indicated that PCD can regulate the immunogenicity of the TME, which influences tumor progression and metastasis, and it is important to elucidate how to avoid chronic inflammation after PCD occurrence in different cell types [18]. For this reason, to better understand the role of PCD in heterogeneous cell populations in TME, which sense and respond to PCD signals, is crucial.

### Apoptosis

**Fig. 2:**
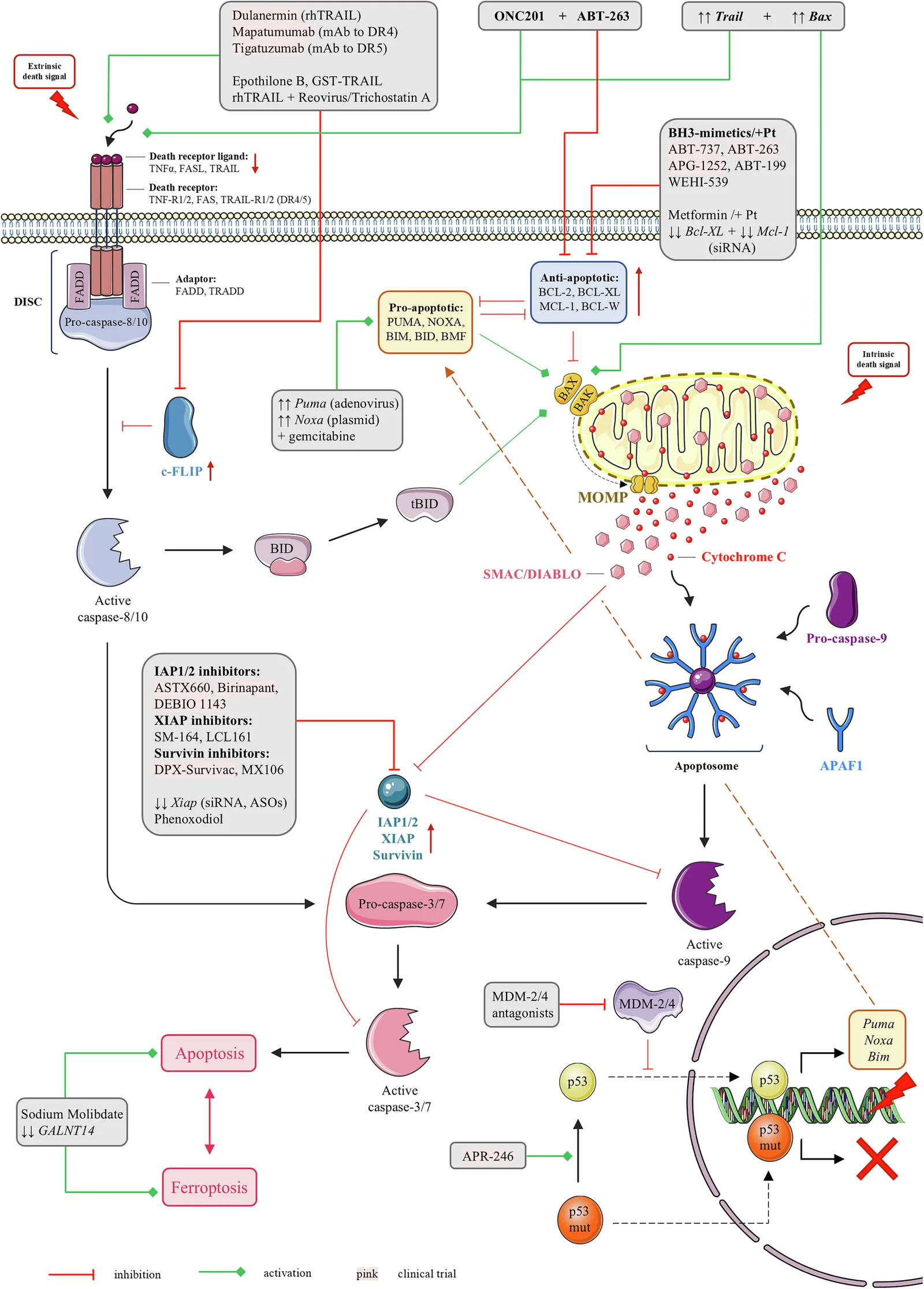
The extrinsic and the intrinsic pathways of apoptosis, along with drug targets. Image provided by Servier Medical Art, licensed under CC BY 4.0.

Since its discovery in 1972 [19], apoptosis has been extensively studied as a fundamental biological process with wide-ranging implications in tissue kinetics. It is characterized by changes in cellular morphology and biochemistry, as well as the activation of cysteine-aspartic proteases known as caspases. Apoptosis can proceed via two major pathways: the extrinsic pathway, which is mediated by death receptors (DRs), and the intrinsic (mitochondrial) pathway [20–22] (Fig. 2).

The intrinsic apoptosis pathway is triggered by various cellular stressors, including DNA damage, growth factor deprivation, oxidative stress, and hypoxia [22]. In response to cell death signals, its activation starts with the formation in the cytosol of the apoptosome complex, which includes cytochrome *c* released from mitochondria, cytosolic proteins APAF1 and pro-caspase-9, as well as dATP. The balance between pro- and anti-apoptotic proteins of the BCL-2 family regulates the release of mitochondrial constituents. Activated within the apoptosome complex, caspase-9 promotes the activation of effector caspases-3/-7 through proteolytic cleavage of their inactive precursors [23–25]. Activated effector caspases subsequently cleave numerous cellular substrates, leading to cell death.

The extrinsic apoptotic pathway is important in maintaining tissue homeostasis, especially in the immune system. This pathway is initiated by the activation of cell surface DRs, including TNFR1/2, Fas (also known as APO-1 or CD95), DR4 (TRAIL-R1), and DR5 (TRAIL-R2). Upon binding to their cognate ligands (TNFα, FasL, TRAIL), these receptors undergo a conformational change to trimerized receptors and subsequently recruit adaptor proteins such as FADD and TRADD and facilitate the formation of the death-inducing signaling complex (DISC), which leads to the autocatalytic activation of initiator caspase-8 (and caspase-10 in certain contexts) [25, 26]. Activated caspase-8 then initiates apoptosis either by direct activation of effector caspase-3 or indirectly through the cleavage of a member of the BH3-only member of the BCL-2 family protein BID to generate truncated BID (tBID), which promotes the activation of proapoptotic BCL-2 family proteins BAX and BAK, resulting in mitochondrial outer membrane permeabilization (MOMP) [27–29]. Consequently, a connection between the extrinsic and intrinsic pathways of apoptosis exists.

Apoptosis is the most extensively studied form of PCD and, for many years, was a primary targeted strategy in anti-cancer therapy. The regulation of apoptosis is essential for tissue homeostasis, maintained by a balance between cell proliferation and cell loss. Disruptions in apoptotic pathways can eventually lead to the accumulation of neoplastic cells. Similar to other solid tumor cells, OC cells develop mechanisms that reduce apoptosis or confer resistance to apoptotic signals by modulating anti-apoptotic or pro-apoptotic components of the cell death machinery [30]. Deregulation of apoptosis-related genes and their products may play a crucial role in ovarian carcinogenesis [31]. Several apoptosis-based therapeutic strategies are currently under investigation and application in the context of OC, with a primary focus on targeting key regulators of both intrinsic and extrinsic apoptotic pathways. Some of them, such as BH3-mimetics, siRNA or shRNA for down- or up-regulation of antiapoptotic BCL-2 proteins in dependence of cancer cell-specific expression pattern, IAPs inhibitors, MDM2/4 antagonists, pro-apoptotic DR agonists, agonistic antibodies to DD receptors, and others will be discussed below (Fig. 2).

The BCL-2 family of proteins, as a central component of the apoptotic signaling network, is associated with tumor development and progression and plays a critical role in the resistance of OC to chemotherapy. Direct changes in the levels of pro- and anti-apoptotic proteins from the BCL-2 family have been the focus of numerous studies, particularly concerning chemoresistance and chemosensitivity in OC [32–34]. In one of the pioneering studies, BCL-2, a key inhibitor of apoptosis, was found to be overexpressed in the OC cell lines and significantly elevated in cisplatin-resistant cells compared with their sensitive counterpart. Furthermore, overexpression of BCL-2 in A2780 OC cells and subsequent drug treatment led to a decrease in the level of pro-apoptotic BAX and tumor protein p53 (TP53), suggesting their involvement in modulating chemotherapy resistance [35]. In EOC tissues, positive staining for BCL-2 significantly correlates with histological type, showing frequent overexpression in endometrioid carcinomas [36, 37]. Conversely, BAX positivity was detected in mucinous tumors specifically at FIGO stages IA-IC [31]. While numerous studies have linked BCL-2 overexpression to chemotherapy resistance, increased malignancy, and poor prognosis [38–40], contrasting data have reported an association between BCL-2 gene expression and enhanced therapy sensitivity, reduced disease progression, and favorable outcomes [41–43]. BCL-2 levels displayed distinct stage-dependent patterns across histological types: they significantly decreased with FIGO stage progression in serous carcinomas but increased in endometrioid carcinomas. A similar, although statistically non-significant, trend was observed in clear-cell and undifferentiated carcinomas. These results demonstrate that BCL-2 expression dynamics depend on histological type in EOC [44]. Importantly, that early observation also revealed a relatively low level of BCL-2 but instead a high level of another anti-apoptotic protein, BCL-XL, and pro-apoptotic BAX in ovarian tissues [45]. Similarly, BCL-2 downregulation was detected in paclitaxel-resistant A2780 cells and in a small cohort of paclitaxel-resistant OC patients, suggesting that this suppression is a characteristic effect of taxane therapy, where BCL-2 acts as an intracellular target for taxanes [46]. Prognostic associations of BAX level in OC also remain controversial. BAX expression is associated with improved disease-free status and OS, with the most significant survival benefit observed in patients co-expressing BAX and BCL-2 [47]. Likewise, a high level of BAX has been reported to correlate with better progression-free survival (PFS) [43]. However, some studies have found that BAX expression predicts an unfavorable clinical outcome, especially in the absence of concomitant BCL-2 expression [48].

Contradictory findings regarding functions of BCL-2 and BAX in OC underscore the complex role of the BCL-2 family in this disease. For example, the paradoxical association of BCL-2 overexpression with improved survival and response to therapy may occur because elevated BCL-2 levels prolong BAX half-life, thereby maintaining a physiologically balanced BCL-2/BAX ratio through a feedback mechanism [47, 49]. Regulation of apoptosis in OC is critically dependent on this ratio. Increased BCL-2 can delay the cell cycle, promoting the accumulation of OC cells in S-phase without altering apoptosis, thereby slowing the growth of cancer cells and improving prognosis [50]. BCL-2 may play a role in maintaining the normal function and integrity of the ovarian surface epithelium during ovulation. A decrease in BCL-2 expression could disrupt this normal ovarian physiology and potentially initiate malignant changes [42]. Notably, AKT-mediated phosphorylation of BAX at Ser184 converts it from a pro-apoptotic protein to an anti-apoptotic one, promoting BH3-mimetic resistance in primary OC cells. This mechanism may explain why increased BAX expression paradoxically correlates with poor prognosis [51]. Finally, histological subtype of OC, disease stage, p53 status, chemotherapy regimens, and methodological variations likely contribute significantly to the observed effects. Therefore, a more nuanced, context-specific understanding of BCL-2’s role and complex interactions between BCL-2 and other proteins is essential for developing effective therapeutic strategies and improving outcomes in OC.

Beyond BCL-2, the antiapoptotic proteins BCL-XL and MCL-1 should also be mentioned in the context of their impact on OC outcomes. Low BCL-XL levels correlate with better PFS in EOC patients [43], while high expression is associated with a shorter relapse-free period after PBC [52]. Overexpression of BCL-XL has been associated with tumor resistance to chemotherapy in in vitro and in vivo models [52]. The level of this protein has a preferential effect on apoptosis in OC cells compared to BCL-2 [50]. MCL-1 transcript expression is significantly elevated in OC, particularly serous and mucinous carcinomas, compared to CCC, and correlates with poor prognosis [53, 54]. Thus, down-regulation of MCL-1 and, especially, BCL-XL could constitute an interesting chemosensitizing approach for OC. In current studies, two strategies targeting anti-apoptotic BCL-2 family proteins are applied in order to induce apoptosis and overcome therapy resistance in OC cell lines: antisense oligonucleotides and RNA interference (siRNA and shRNA), and BH3-mimetics (small molecules that mimic BH3-only proteins to bind and inhibit these anti-apoptotic proteins) [33]. Despite the siRNA-mediated suppression of BCL-XL in combination with cisplatin promoting apoptosis in chemoresistant OC cells, a subset of cells can survive and later re-proliferate. Better results might be achieved when the concomitant inhibition of both BCL-XL and MCL-1 in combination with cisplatin was utilized because it led to complete eradication of the tumor cell population and prevented subsequent relapse [55]. A combination of BH3-mimetic ABT-737 (suppresses BCL-XL/BCL-2) with RNA interference or platinum derivatives (directly or indirectly suppresses MCL-1) can also evoke pronounced apoptosis in vitro and ex vivo. Additionally, the up-regulation of NOXA (a BH3-only protein), which is induced by platinum compounds, may be useful as it is required for the sensitization of OC cells to ABT-737 [56]. The antidiabetic drug metformin dose-dependently reduced levels of BCL-2, BCL-XL, and MCL-1 in EOC cell lines and synergistically enhanced cisplatin-induced apoptosis [57]. A phase I clinical trial combining metformin with carboplatin/paclitaxel in patients with EOC has demonstrated safety and feasibility [58]. Collectively, these findings underscore the therapeutic promise of co-targeting key anti-apoptotic BCL-2 family members, particularly BCL-XL and MCL-1, in combination with conventional chemotherapy for treating chemoresistant and recurrent OC.

The IAP (inhibitor of apoptosis protein) family plays a crucial role in the suppression of apoptosis alongside the anti-apoptotic BCL-2 proteins. IAPs directly inhibit caspases, the key executioner enzymes of apoptosis. Overexpression of the IAP family is common in OC and correlates with poor prognosis and chemotherapy resistance. Specifically, XIAP (X-linked IAP) is highly expressed in EOC, and its downregulation induces apoptosis, particularly in cisplatin-sensitive cells [59]. Elevated XIAP expression correlates with platinum resistance and poor prognosis in CCC patients. Accordingly, siRNA-mediated XIAP silencing sensitized CCC patient-derived cells to cisplatin [60]. Antisense targeting of XIAP triggered apoptosis in OC cells in vitro and increased survival in OC mouse models in vivo [61]. Survivin, an IAP family member, is overexpressed in > 50% of EOC and correlates with advanced FIGO stage, relapses, and poor survival. Survivin ablation inhibited primary tumor growth and metastasis in an orthotopic OC mouse model. Thereby, reducing XIAP or survivin levels may restore OC sensitivity to chemotherapy and improve treatment response, especially when combined with standard therapy. Indeed, the selective survivin inhibitor MX-106 induced apoptosis in paclitaxel-resistant OVCAR3 cells, suppressed invasion and migration in OC SKOV3 and OVCAR3 cells, and reduced tumor growth/metastasis in an orthotopic OC mouse model [62]. Respectively, phenoxodiol, an isoflavone derivative, inhibited XIAP, restoring sensitivity in docetaxel-resistant OC cells and demonstrating therapeutic potential for refractory OC [63].

Similar to intrinsic apoptosis dysregulation, the extrinsic apoptotic pathway is frequently impaired in OC, contributing to chemoresistance and tumor progression. Mechanisms include downregulation or modification of DRs and dysregulation of downstream signaling, collectively evading apoptosis [64]. Hence, reactivating extrinsic apoptosis could be a rational treatment option to bypass the typical resistance that cancers foster against internal stress signals. Current approaches focus on pro-apoptotic DR agonists such as recombinant human TRAIL (rhTRAIL) and agonistic antibodies targeting DR4/DR5. Expression of Fas and TRAIL in OC is associated with lower tumor grade and improved PFS [65]. Reduced Fas expression correlates with adverse features: it is lower in chemoresistant tumors compared to chemosensitive ones [43] and in OC patients versus healthy women [66]. Cisplatin treatment increases Fas level in both sensitive and resistant OC cells but elevates FasL only in sensitive cells [67]. Fas positivity was linked to low tumor grade and serous histotype, while associating FasL expression with advanced stage [68]. This elevated FasL expression may contribute to immune evasion by inducing apoptosis of Fas-expressing tumor-infiltrating lymphocytes [69]. Approximately 40% of EOC patients exhibit elevated c-FLIP_L_ (long form of c-FLIP) levels, which bind FADD to block apoptosis [70]. Significantly, upregulation of c-FLIP induces cisplatin resistance in OC cells, while its downregulation reverses resistance and restores cisplatin-induced apoptosis [71]. These findings establish c-FLIP blocking as a possible therapy for cisplatin-resistant EOC. The death ligand TRAIL is associated with improved OS in OC [72]. Combining recombinant glutathione-S-transferase (GST)-TRAIL fusion protein with chemotherapy synergistically enhances apoptosis in resistant OC cell models [73, 74]. These findings are consistent with those that found that rhTRAIL monotherapy induced apoptosis in 50% of OC cell lines, but its combination with chemotherapeutic agents produced strong synergy and overcame TRAIL resistance [75]. In addition, resistance to TRAIL was associated with either the absence of DR4 expression or an elevated level of c-FLIP_S_ (the short form) and a cleavage product of c-FLIP_L_. While TRAIL alone had minimal effect on DR4/DR5, its combination with cisplatin synergistically increased their surface expression in OC cells [76]. Several potential therapeutic agents have been shown to induce the extrinsic apoptosis pathway, either as monotherapy or in combination with rhTRAIL, such as epothilone B (a microtubule-disrupting agent) [77], reovirus (an oncolytic virus that selectively targets cancer cells) [78], trichostatin A (an HDAC inhibitor) [79], and the DR5-specific agonistic monoclonal antibody TRA-8 [80]. Preclinical evidence supporting TRAIL pathway agonists for OC treatment has progressed to phase I/II clinical trials. Agents under investigation include dulanermin (rhTRAIL) [81]; mapatumumab, a high-affinity agonistic monoclonal antibody targeting DR4 [82]; and CS-1008 (tigatuzumab), a humanized anti-DR5 antibody, evaluated with carboplatin/paclitaxel (NCT00945191). Consequently, TRAIL-based therapy, including recombinant TRAIL proteins or anti-DR4/5 antibodies, when combined with chemotherapy, may overcome resistance and effectively activate the extrinsic apoptosis pathway. The concept of simultaneous activation of extrinsic and intrinsic apoptotic pathways represents a promising therapeutic approach for OC therapy, aiming to overcome tumor resistance by targeting cancer cells with dual apoptotic signals, leading to more effective and comprehensive treatment. Combining ONC201 (TIC10), which upregulates ligand TRAIL and its receptor DR5, with the BH3 mimetic ABT-263 synergistically induced strong apoptosis in OC cells [83]. Similarly, it was demonstrated that combined TRAIL and BAX gene therapy significantly improved survival in vivo compared to either agent alone [84]. Another significant effective strategy in combating cancer may be the cross-induction of multiple cell death pathways, causing a more potent, synergistic cell death response that makes it harder for tumors to survive. For example, it was demonstrated that sodium molybdate triggers both apoptosis and ferroptosis in OC models in vitro and in vivo [85]. Additionally, targeting GALNT14, which is upregulated in cisplatin-resistant cells, enhanced apoptosis and ferroptosis, revealing a potential strategy to reverse cisplatin resistance in OC [86]. Recently, the contribution of non-coding RNAs (ncRNAs) (mainly including microRNAs (miRNAs), long noncoding RNAs (lncRNAs), and circular RNAs (circRNAs)) to apoptosis dysregulation in OC and their potential as therapeutic targets and diagnostic biomarkers has been reviewed in detail [87].

It is worth noting that ncRNAs regulate gene expression at various levels and can promote or inhibit apoptosis, thus modulating the balance between cell death and survival in response to chemotherapy. There are several miRNAs involved in response to PBC or other apoptosis-inducing agents—miR-509-3p, miR-630, and miR-146a-5p. Overexpression of miR-509-3p can downregulate the expression of XIAP, increase sensitivity to cisplatin, and inhibit the proliferation of EOC cells [88]. It has also been shown that the miR-630 inhibitor decreased cell proliferation and enhanced the sensitivity of therapy-resistant SKOV3 cells to paclitaxel, probably by targeting APAF-1 [89]. In OVCAR3 and SKOV3 cells, miR-146a-5p enhances the therapeutic effects of cisplatin through suppression of *XIAP*, *BCL2L2*, and *BIRC* genes [90]. An oncogenic role was suggested for many lncRNAs (MALAT1, TP73-AS1, ABHD11-AS1, HOTAIR) because their silencing led to reduced cell proliferation and increased apoptosis. For some lnсRNAs have been shown to suppress apoptosis through interaction with miRNAs. For example, DUXAP8 prevents apoptosis by suppressing miR-590-5p. LINC00152 can be a competitive endogenous RNA for miR-125ba, thus influencing apoptosis. Binding HAGLROS to miR-26b-5p or NEAT1 to miR-34a-5p can also suppress apoptosis. These examples depict the complex role and notable impact of lncRNAs in apoptosis modulation in OC. Similarly, circRNAs play critical roles in regulating apoptosis by interacting with miRNAs in OC. Circular RNA hsa_circ_0078607 enhances apoptosis by increasing Fas expression through miR-518a-5p [91]. Interestingly, silencing of circRNAs could restore apoptosis in OC cells. Knockdown of hsa_circ_0013958 elevated the cell apoptotic rate and suppressed proliferation, migration, and invasion of OC cells [92]. Remarkably, the high specificity of detection methods for ncRNAs and their tissue-specificity allows testing them as biomarkers for a disease state or a prognostic marker for disease outcome in the majority of clinical trials. That can be very critical for early detection of OC, which is often not diagnosed until an advanced stage. Nevertheless, ncRNAs can be considered as promising candidates for targeted therapy. It is essential to continue clinical trial investigations for evaluation of it as an effective treatment for OC.

In summary, dysregulation of apoptosis is a fundamental driver of OC development and progression, allowing tumor cells to evade cell death. This acquired resistance to apoptosis poses a major therapeutic challenge. Therefore, targeting key components of both the intrinsic and extrinsic apoptotic pathways (through monotherapy or combination therapy) and inducing multiple cell death pathways represent critical, actively pursued strategies for developing new OC treatments. The above-described data highlight the need for further preclinical and clinical studies on the co-activation of apoptosis pathways in OC therapy.

### Autophagy

**Fig. 3:**
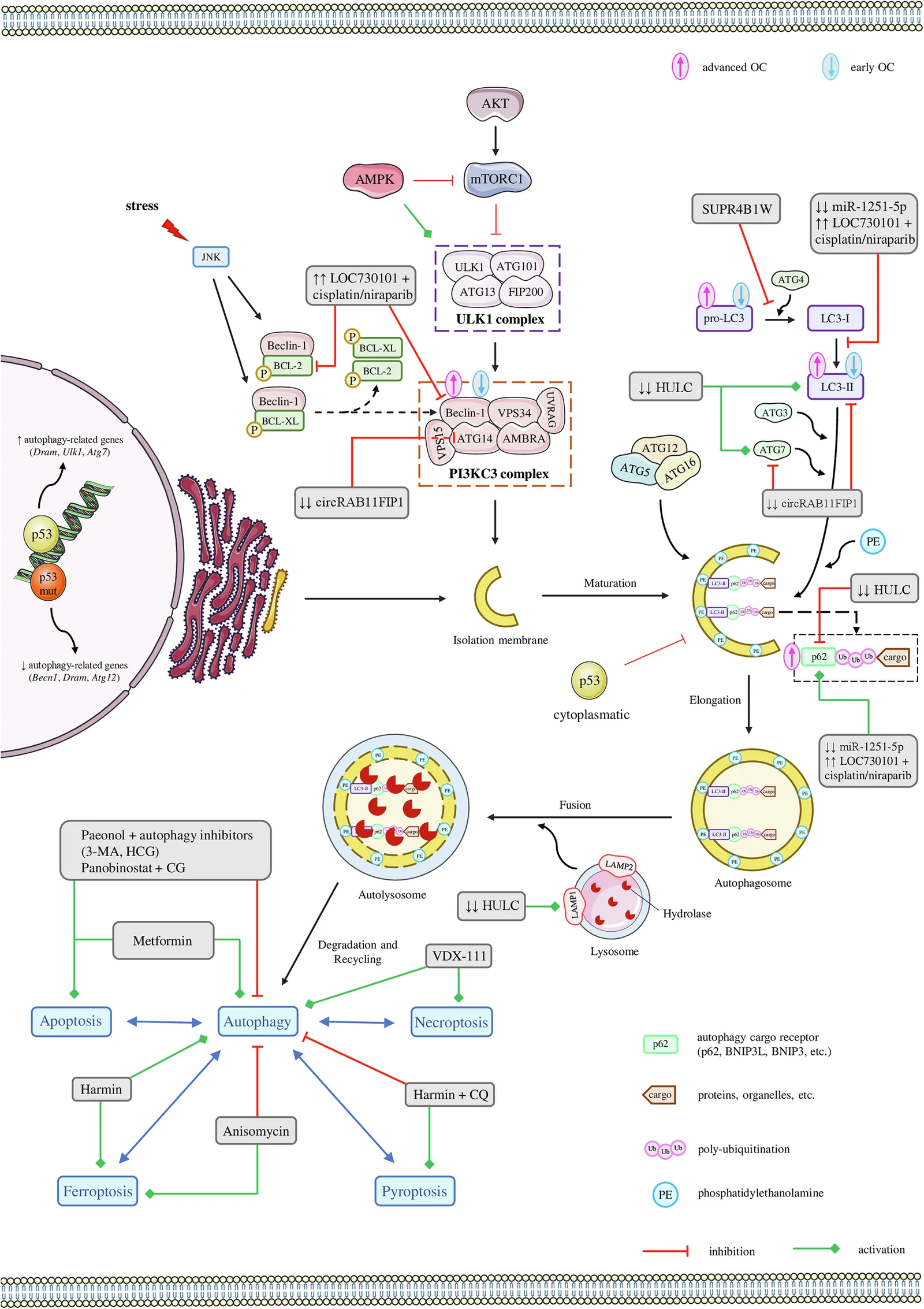
Basic steps of the autophagy pathway and drugs targeting autophagy-related proteins. Image provided by Servier Medical Art, licensed under CC BY 4.0.

Autophagy is a regulated process that maintains cellular homeostasis and organelle integrity by the delivery of various cellular cargoes to lysosomes for degradation and recycling in normal physiological conditions. Christian de Duve was the first to hypothesize the existence of cellular machinery that transported cytosolic constituents to lysosomes for digestion and created the term ‘autophagy’ in 1963 [93]. In the last decades, a panel of autophagy-related gene (ATG) products (more than 40 in yeast, of which approximately one-half are mammalian homologs) involved in the formation of the autophagosome, which degrades cellular cargo through lysosomal hydrolase activity, has been identified (Fig. 3).

It is now widely accepted that autophagy plays a suppressive role in tumor initiation, while the knockout mice for certain autophagy genes (*BECN1, UVRAG, Atg5, Atg7*, and others) are predisposed to different types of cancers (lymphoma and liver cancer) [94, 95]. Indeed, *BECN1*, which is associated with the formation of the autophagosome, is often deleted in breast and OC [96]. The TCGA program has shown that almost all tested HGSOC (489 patients) possess the tumor suppressor gene *TP53* mutation [97]. It is known that wild-type p53 under stress can promote autophagy, while mutant p53 can inhibit autophagy by repressing expression of several autophagy-related proteins and enzymes, including Beclin-1, DRAM, and ATG12 [98]. While autophagy functions at multiple levels to maintain the suppression of tumorigenesis and protect genome stability, for example, by removing damaged mitochondria (mitophagy), which regulates the reactive oxygen species (ROS) level contributing to DNA damage and possessing tumor-initiating capacity [99], some evidence suggests that in established tumors, autophagy is required to support cellular homeostasis of cancer cells and tumor progression. Investigation of the expression of two autophagy-related proteins, Beclin-1 and LC3 (also known as MAP1LC3B), revealed that in FIGO stages I and II patients, decreased levels of their expression may be related to tumorigenesis and development of EOC [100]. The high level of p62 (also known as SQSTM1) and LC3, and weak BECN1 expression was observed in invasive tumor segments of advanced stages of EOC [101]. Investigation of patients with ovarian CCCs revealed high LC3 expression and showed a lower response to the PBC [102]. Among patients with post-chemotherapy effusions, increased expression of p-mTOR was an independent predictor of poor PFS [103]. Usually, during cancer progression, hypoxia is an indispensable condition that evokes ROS production, which damages organelles and induces autophagy, which permits cancer cells to survive. A high level of mitophagy factor BNIP3 results in resistance to PBC in in vitro cisplatin-resistant models and in OC patients’ samples due to enhanced mitophagy [104]. Thus, in advanced stages of cancer cell development, autophagy plays a pro-survival role and may contribute to chemoresistance, and the inhibition of autophagy can reverse chemoresistance in OC cells [105, 106].

Under certain conditions, excessive autophagy can mediate cell death, which can be independent of other forms of PCD or accompanied by various modes of death (apoptosis, necroptosis, ferroptosis, and pyroptosis). For example, the clinically approved drug metformin induced apoptosis in a concentration-dependent manner and also promoted autophagy in SKOV3 cells [107]. The antibiotic anisomycin, by suppressing glutathione (GSH) metabolism and downregulating the expression of ATF4 in OC cells, can induce ferroptosis and autophagy [108]. Treatment of a panel of OC cell lines with VDX-111 (necroptotic inducer) causes a dose-dependent loss of cell viability by induction of changes in cell signaling related to autophagy and necroptosis [109]. Interestingly, plant alkaloid harmine enhances autophagy in SKOV3 cells by modulating the PI3K/AKT/mTOR/FOXO3 signaling pathway, resulting in reduced cell proliferation. Furthermore, the same compound was found to induce ferroptosis in these cells by elevating the level of Fe^2+^ and malondialdehyde (MDA), while concurrently decreasing the level of antioxidants such as GSH and superoxide dismutase (SOD). Additionally, harmine triggered pyroptosis through the upregulation of NLRP3, caspase-1, IL-18, and GSDM-D. This study further demonstrated that autophagy can positively regulate ferroptosis while negatively regulating pyroptosis [110]. Importantly, despite the existence of the examples described above and many others, the detailed mechanisms of such interactions are still unknown and remain to be elucidated.

Recent studies have shown that in OC cells, ncRNAs can modulate the formation of autophagosomes, which affect tumor progression and chemoresistance. For instance, miR-1251-5p triggers cell proliferation and autophagy by suppressing the expression of the tumor suppressor gene, *TBCC*, and is highly expressed in human OC cells and tissues [111]. The lncRNA HULC, highly upregulated in hepatocellular carcinomas, has been implicated in the progression of multiple malignancies, including EOC. HULC promotes progression of OC primarily through the downregulation of *ATG7*, thereby suppressing autophagic activity. Moreover, the downregulation of HULC was found to suppress OC invasion and migration, which correlates with alterations in integrin ITGB1 levels and enhanced apoptosis [112]. These results suggest that inhibiting HULC could be an effective genetic therapy for OC. In contrast, overexpression of lncRNA LOC730101 significantly increased apoptosis induced by PBC and PARP inhibitors, whereas its silencing reduced the sensitivity of OC cells to these treatments. Under chemotherapy conditions, LOC730101 overexpression was associated with a decrease in the levels of the anti-apoptotic protein BCL-2 and the autophagy marker LC3-II, alongside an accumulation of the autophagy substrate p62. Collectively, these findings suggest that elevated LOC730101 expression may potentiate drug sensitivity in OC cells by suppressing protective autophagy and enhancing apoptotic pathways [113]. In recent years, an expanding body of research has identified numerous circRNAs that function as molecular sponges for miRNAs or RNA-binding proteins, regulate alternative splicing and gene expression, and contribute to various aspects of tumorigenesis [114]. Among these, circRAB11FIP1 is significantly upregulated in EOC tissue samples. Furthermore, its overexpression led to enhanced autophagy in A2780 OC cells, promoting both proliferation and invasion of OC [115], suggesting that circRAB11FIP1 may serve as a potential diagnostic and therapeutic biomarker for EOC. The modulation of ncRNAs may be beneficial for OC treatment by inhibiting protective autophagy or facilitating autophagic cell death.

Currently, many substances, such as naturally occurring alkaloids, flavonoids, phenols, glycosides, esters, and volatile oils, have been tested for modulation of autophagy [116]. Some natural products (silybin, elemene, quercetin) activate autophagic cell death, while others (ginsenoside Rg1, β-asarone, fisetin) inhibit it. Interestingly, several autophagy inhibitors act synergistically with various OC drugs. Thus, chloroquine and panobinostat, a histone deacetylase inhibitor, induced apoptosis and suppressed autophagy in OC cell lines [117]. Paeonol, a phenolic acid compound with antitumor activity, has been found to initiate apoptosis in a dose-dependent manner and also activate cytoprotective autophagy in A2780 and SKOV3 OC cells by inhibiting the AKT/mTOR signaling pathway and contributing to chemotherapy resistance in these cells. Combining treatment with paeonol and autophagy inhibitors exerted significant synergistic effects in reducing tumor cell viability and promoting apoptosis both in vitro and in vivo. These findings suggest that co-administration of paeonol with autophagy inhibitors may potentiate its antitumor efficacy for the treatment of OC [118].

Thus, depending on the cellular context and whether the therapeutic goal is to inhibit or induce autophagy, an efficacious anticancer therapy can be developed, which can stimulate the death of OC cells. It is important to note that tumor cells with high or low autophagy levels have separate interactions with immune cells, especially macrophages, and the autophagy-related signature can be useful for predicting prognosis and the efficacy of immune checkpoint inhibitor therapy in OC patients [119]. Further investigations are required to clarify the role of autophagy in cancer development and treatment, and how the autophagy pathway can be regulated and targeted for cancer prevention and therapy.

### Ferroptosis

**Fig. 4:**
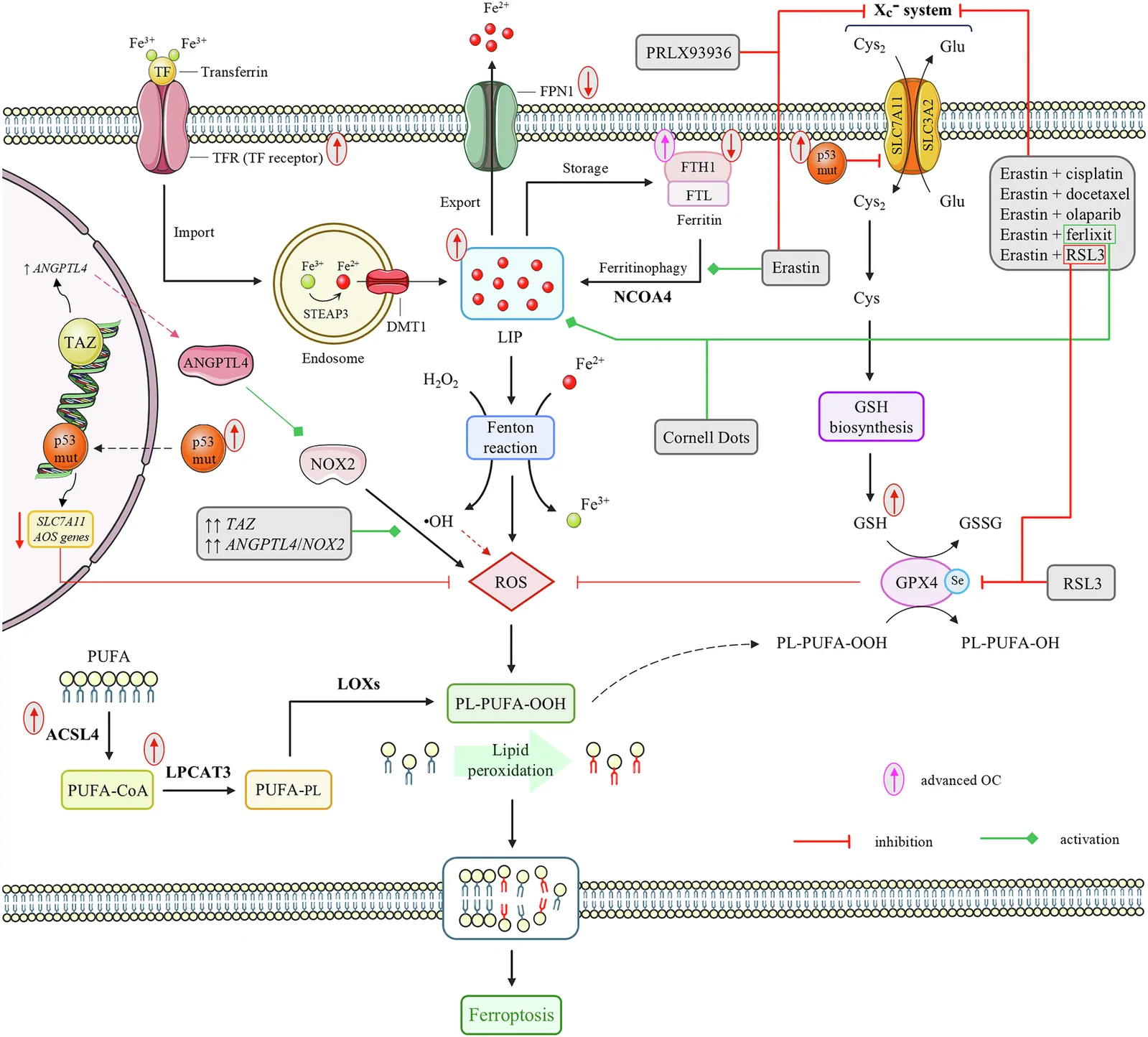
Ferroptosis pathway and potential therapeutic targets. Image provided by Servier Medical Art, licensed under CC BY 4.0.

Iron is an essential trace element critical for cell proliferation and growth, serving as a cofactor of many enzymes involved in the regulation of DNA synthesis and repair, redox reactions, and heme synthesis [120]. In 2012, a new type of PCD, ferroptosis, was described and found to be iron-dependent [121]. Ferroptosis differs from other types of PCD, such as apoptosis, necrosis, and autophagy, in morphology and biochemistry. Morphologically, ferroptotic cells are characterized by decreased mitochondrial volume, increased mitochondrial bilayer membrane density, reduced or absent mitochondrial cristae, disruption of the OMM, absence of chromatin condensation, and apoptotic body formation. There are no biochemical signs of apoptosis—cytochrome *c* release from mitochondria, pro-caspase-3 processing, and PARP1 cleavage [122]. Ferroptosis is regulated by multiple molecular mechanisms linked to iron metabolism, ROS production, and lipid metabolism. Mechanistically, ferroptosis is triggered by excess intracellular iron, which drives the Fenton reaction and the generation of hydroxyl radicals (•OH) from H_2_O_2_. If the antioxidant mechanisms are insufficient or compromised, the •OH and other ROS may attack polyunsaturated fatty acids of cellular membranes, which are extremely susceptible to ROS, and induce lipid peroxidation that damages the cell membrane and triggers ferroptosis (Fig. 4).

Iron cellular homeostasis is maintained by several regulatory mechanisms, including iron uptake, storage, metabolism, and secretion [123]. Transferrin receptors (TFRs) mediate the import of insoluble ferric iron (Fe^3+^) by internalizing the transferrin-iron complexes through receptor-mediated endocytosis. STEAP3 metalloreductase reduces ferric iron (Fe^3+^) to ferrous iron (Fe^2+^) in endosomes, and DMT1 (SLC11A2) mediates the release of Fe^2+^ from the endosome into the cytoplasm, where it can be stored in the labile iron pools (LIPs) or the iron-storage protein ferritin (FTL, FTH1). Ferritinophagy, which is an autophagic process, promotes iron release from ferritin when iron is needed. Iron from LIPs may be used for generating iron-dependent protein complexes, iron-sulfur clusters, and heme or, as an enzymatic cofactor, used in metabolic pathways [124]. The excess of Fe^2+^ can be excreted from the cell by the iron efflux pump ferroportin 1 (the iron efflux pump, also known as FPN1, SLC40A1) or can be stored within the ferritin complex.

It has been proposed that iron-induced oxidative stress derived from retrograde menstruation may initiate carcinogenesis in serous, endometrioid, and CCCs in situations of unbalanced antioxidant defenses [125]. Iron deposits have been identified within the fallopian tube of patients diagnosed with serous epithelial OC [126]. Iron was measured to be ~100 mM in endometriotic cysts and was associated with lipid peroxide [127]. The genes involved in alterations in the iron pathway in OCs (*IRP1, FTH1, FTL, HAMP, IRP2, NCOA4, TF, TFRC, DMT1, FPN1*) were described and presented evidence implicating them in OC biology [128]. Thus, reduced expression of FTH1 was identified in OC and associated with poor patient outcome [129], but its expression was elevated in metastatic OC [130]. Modulating any of the processes involved in iron metabolism could be a feasible targeting strategy for OC treatment. The alterations in the expression of genes involved in the iron pathway may induce iron overload in cancer cells and make them more vulnerable to ferroptosis induction. This hypothesis was tested in tumor-initiating cells (TICs, “cancer stem cells”), which represent a small pool of treatment-refractory cells that contribute to OC drug resistance and recurrence. TICs have an apparently higher level of TFR1 (the iron importer) and a lower level of ferroportin, resulting in the excessive accumulation of intracellular iron and demonstrating enhanced sensitivity to erastin, a ferroptosis-inducing agent, which inhibits system Xc- and activates mitochondrial porins, VDAC [131]. System Xc- is an amino acid antiporter that plays a pivotal role in the GSH system by mediating the uptake of cystine, which, upon reduction to cysteine, serves as the rate-limiting substrate for GSH synthesis. Ferroptosis can be triggered by inhibition of system Xc- or glutathione peroxidase 4 (GPX4) and the depletion of GSH as a major antioxidant system, which participates in neutralization of ROS and prevention of lipid peroxidation. GSH is required for GPX4 to eliminate oxidized phospholipids, and system Xc- is critical to GSH production. If system Xc- was inhibited by sorafenib, it caused GSH depletion and subsequent inactivation of GPX4 and accumulation of toxic lipid peroxides, leading to ferroptosis. It allows us to propose that ferroptosis-inducing agents (FINs) can be used as a potential treatment of OC with an altered iron import/export expression system and an iron-addicted profile, either alone or in combination with conventional therapies.

The ability of erastin to inhibit cell growth by inducing ferroptosis in OC cell lines and OC tumor xenografts has already been tested in combination with cisplatin [132]. Erastin appeared to maximize the therapeutic effects of cisplatin while minimizing side effects in OC both in vitro and in vivo. Erastin also synergizes with cisplatin to overcome OC cells’ resistance to the latter [133]. In fact, erastin can also overcome docetaxel resistance and augment docetaxel efficacy in OC by inhibition of ABCB1, which is overexpressed in cancers and contributes to reduced intracellular accumulation of chemotherapeutic agents [134]. Olaparib, a clinically used PARP inhibitor, dramatically promotes ferroptosis in BRCA wild-type OC cells and, in combination with erastin, synergistically sensitizes BRCA-proficient OC cells to PARP inhibition [135]. Erastin induces NCOA4-mediated ferritinophagy and mitochondrial dysfunction and triggers ferroptosis in the HEY OC cells with a high intracellular labile iron pool but not in COV318 cells with a low baseline pool. The combination of erastin and ferlixit, an iron compound used to treat patients suffering from anemia, sensitizes COV318 cells to erastin and could overcome ferroptosis chemoresistance in OC cells [136]. Inhibition of GPX4 can be applied for the induction of ferroptosis by using (1S,3 R)-RSL3, which covalently binds to the selenocysteine active site [137]. Erastin and RSL3 demonstrated cytotoxic effects in K-Ras mutant human OC cells, OVCAR-8 and NCI/ADR-RES [138]. Thus, FINs, like erastin and RSL3, allow the selective treatment of tumors with higher iron levels, as they may employ different mechanisms of cell death and target resistant tumors. PRLX93936, an analog of erastin, has been tested in clinical trials. It synergized with cisplatin, inducing lipid peroxidation and Fe^2+^ production for promoting ferroptosis in non-small cell lung cancer cells [139]. Another substance that has reached clinical trials and affects iron metabolism is ELU001 (a C’Dot drug conjugate). C’Dots, or Cornell Prime Dots, are ultrasmall fluorescent silica core-polyethylene glycol (PEG) shell (core-shell) hybrid nanoparticles. They can bind iron in the extracellular environment and, after ingestion, lead to an increase in the level of intracellular iron and cause an accumulation of ROS and cell death via ferroptosis. C’Dots have been shown to lead to tumor regression in in vivo mouse xenograft models [140], and, after having been proven safe and effective in three previous diagnostic human clinical trials, the C’Dot drug conjugate, ELU001, received approval for the first therapeutic trial as a treatment for patients with advanced, recurrent, or refractory cancers overexpressing folate-receptor alpha, including ovarian, breast, and lung cancers [141].

Abnormal lipid metabolism is an important driver of ferroptosis. Free polyunsaturated fatty acids (PUFAs) undergo esterification into membrane phospholipids (PLs) and act as substrates for lipid synthesis. They are highly susceptible to lipid peroxidation and thus crucial for ferroptosis induction [142, 143]. Two enzymes, ACSL4 (acyl-CoA synthetase long-chain family member 4) and LPCAT3 (lysophosphatidylcholine acyltransferase 3), are actively engaged in PLs biosynthesis and remodeling. Suppression of *ACSL4* expression increases ferroptosis resistance, whereas overexpression of *ACSL4* restores ferroptosis sensitization in cancer cells [144]. In OC tissues, ACSL4 was upregulated, and high ACSL4 expression predicted a worse prognosis and sensitized OC cells to erastin- and RSL3-induced ferroptosis [145]. High expression of *LPCAT3*, which is involved in PLs’ remodeling to achieve appropriate membrane lipid fatty acid composition, was also associated with poor prognosis in OC, and it was integrated into a prognostic signature of OS patients [146].

There are two known ways of intracellular lipid peroxidation: non-enzymatic and enzymatic. In non-enzymatic hydroxyl lipid peroxidation, the hydroxyl radicals generated by the Fenton reaction oxidize PUFAs to lipid hydroperoxides. In enzymatic lipid peroxidation, the direct oxidation of PUFAs to various types of fatty acid hydroperoxides occurs in reactions catalyzed by lipoxygenase (LOX), an iron-containing enzyme whose inhibitors can prevent ferroptosis in oxidative damage conditions. The accumulation of fatty acid hydroperoxides in the cell membrane can cause structural changes in the fluidity of the cell membrane as well as an increase in membrane permeability, leading to loss of membrane integrity. Overexpression of LOX-5, LOX-12, and LOX-15 was shown to sensitize cells to ferroptosis, and LOX inhibitors effectively act as an antioxidant to protect HEK293 cells from lipid peroxidation [147]. However, its role in ferroptosis in OC cells needs to be tested. Moreover, membrane electron transport proteins and the NADPH-generating pentose phosphate pathway also contribute to ROS generation and lipid peroxidation in ferroptosis. NADPH oxidases (NOXs), which catalyze the formation of superoxide, impact the iron-dependent accumulation of lipid peroxidation. In OC cells, the activity of NOX2 can be managed by the gene *ANGPTL4*, which is a target of TAZ, and the knockdown of *NOX2, TAZ*, and *ANGPTL4* reduced erastin-induced lipid peroxidation in CAOV2 OC cells [148]. This means that ferroptosis-induced therapies may be potentially effective against ovarian tumors with TAZ activation.

Current findings suggest that various ncRNAs, including miRNAs, lncRNAs, and circRNAs, can regulate ferroptosis in OC cells, and this growing body of evidence was analyzed in detail [149]. Below, a few examples are highlighted to describe how ncRNAs can influence ferroptosis. Thus, erastin- and RSL3-induced ferroptosis in OC cells can be lowered by upregulation of miR-424-5p, which directly binds to the 3’-UTR of ACSL4 and suppresses the expression of its gene [145]. Erastin-induced ferroptosis can also be inhibited by lncRNA TPT1-AS1, which regulates GPX4 transcription via CREB1 and interacts with RNA-binding protein KHDRBS3 for the regulation of CREB1 [150]. Knockdown of circSnx12 reduced cell viability and enhanced ferroptosis in cisplatin-resistant OC cells due to its inability to act as a molecular sponge for miR-194-5p, which suppresses SLC7A11 expression [151]. In addition to the impact of ncRNAs on proteins involved in the disruption of amino acid, ROS, and lipid metabolism, which trigger ferroptosis, it is important to note that the level of ncRNAs varies between subtypes of OC and can be used in the future as clinically relevant markers that can enhance stratification, diagnosis, and prognosis of patients with OC. Thus, multiple investigations have indicated that the induction of ferroptosis in OC cells may be a viable strategy in combination therapies to improve response rate and overcome the resistance of OC.

### Necroptosis

**Fig. 5:**
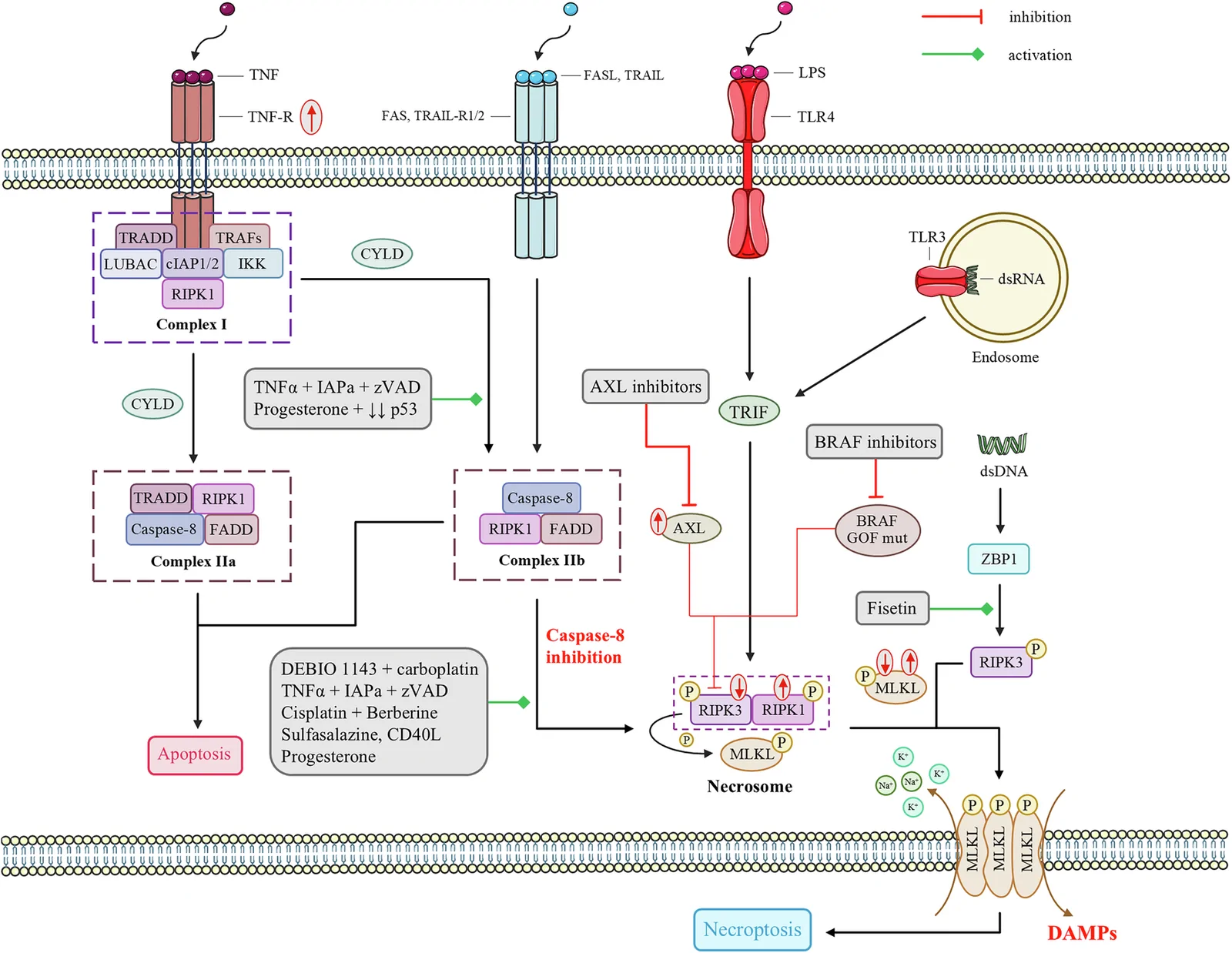
Necroptosis pathways regulated with potential drugs. Image provided by Servier Medical Art, licensed under CC BY 4.0.

Almost 20 years ago, an alternative form of regulated cell death (RCD) in the absence of intracellular apoptotic signaling was described and termed necroptosis [152]. Necroptosis culminates in lytic necrosis of the cell. It is triggered under circumstances when the normal execution of apoptosis cannot occur and often presents the mode of caspase-independent cell death, which may be activated via a block of apoptotic caspase-8 (Fig. 5).

Such inhibition may arise from an inherited mutation, virally encoded inhibitors, the host factor cFLIP, reduced expression of caspase-8, or its pharmacological inhibition. Necroptosis can be initiated with extracellular or intracellular signals. Among them are extracellular (TNF, TRAIL, FASL, LPS, dsRNA) and intracellular (dsDNA) molecules. Upon binding of ligands to corresponding receptors or other targets (for example, Z-DNA binding protein 1 (ZBP1) binds to dsDNA), the unique signalosome complexes are assembled, and if the magnitude of the signal is sufficient to suppress the survival downstream pathways, it promotes the formation of a pro-necroptotic multi-protein signaling complex (“necrosome”, also known as complex IIb). Necrosome consists of activated (phosphorylated) RIPK1 and RIPK3 heterodimers. The formation of RIPK1-RIPK3 heterodimers also promotes RIPK3 homodimerization. These homodimers can act as docking stations for the recruitment of the pseudokinase mixed-lineage kinase domain-like (MLKL), the pore-forming executioner of necroptosis. RIPK3 can directly phosphorylate (Thr357 and Ser358) and activate MLKL [153, 154], triggering its conformational change leading to the formation of higher-order MLKL oligomers, which can bind to membrane phosphatidylinositol lipids and cardiolipin [155]. Membrane-bound MLKL oligomers form transmembrane cation channels, which are permeable to Mg^2+^, Na^+^, and K^+^ but not to Ca^2+^ and trigger MLKL-induced membrane depolarization as well as cell death that culminates in the release of intracellular content [156].

Among the various stimuli that trigger necroptosis, TNF-α is the most intensively investigated and considered a prototype of regulated necrotic cell death. The mechanism of its action was described earlier [157–159]. Since the elimination of tumor cells via induction of cell death is a goal of cancer therapy, to enhance the efficacy of cytotoxic therapy, several compounds have been investigated for their ability to induce necroptosis in OC cells. TNF-α in combination with IAP antagonists and pancaspase inhibitor zVAD triggers necroptosis in apoptosis-resistant OVCAR3 cells and in cisplatin-resistant patient-derived tumor cells [160]. CD40L (the cytokine from the TNF family) also induces RIP1-dependent necroptosis-like cell death in LGSOC cells [161]. Sulfasalazine, a drug used to treat rheumatoid arthritis, which may suppress cystine-glutamate exchange (xCT), induces necroptosis in cisplatin-resistant SKOV3 cells [162]. However, the combination of cisplatin with berberine, a compound extracted from various herbs, significantly inhibits the proliferation of OVCAR3 and primary OC cells from patients by inducing apoptosis and the RIPK3-MLKL necroptotic pathway [163]. Fisetin, a natural flavonoid, reduced cell growth of OC cell lines by inducing apoptosis and ZBP1-dependent necroptosis simultaneously via the RIPK3/MLKL pathway [164]. Progesterone (P4) could induce tumor cell necroptosis through the TNF-α/RIPK1/RIPK3/MLKL pathway in Trp53-/- mouse oviduct epithelium and in immortalized human p53-deficient fimbria epithelium, and a progesterone receptor (PR) antagonist attenuated P4-induced necroptosis. This suggests that necroptosis can occur in p53-defective oviduct epithelium as an in vivo protection mechanism against OC occurrence, but PRs are largely deregulated or non-expressed in EOCs [165].

Expression of key necroptosis regulators is also generally downregulated in OC cells, indicating that cancer cells may evade necroptosis to survive [166]. Thus, escape from necroptosis is prevalent across cancer types according to a cell-based necroptosis sensitivity screen of 941 cancer cell lines. Genome-wide bioinformatic analysis revealed resistance to necroptosis and loss of RIPK3 expression. The progressive loss of RIPK3 expression occurs during tumor growth, both in patient tumor biopsies and tumor xenograft models [167]. For the Chinese population of EOC patients, the association of genotypes of RIPK1 tag SNPs, increased plasma levels of RIPK1 protein, and reduced RIPK1 mRNA expression in white blood cells was observed along with increased risk for early onset of EOC [168]. RIPK1 knockout significantly suppressed tumor growth and greatly attenuated cisplatin’s antitumor activity in human OC A2780 cell line xenograft nude mice [169]. The low MLKL level correlated with decreased OS and decreased disease-free survival in 153 individuals with resectable primary OC [170]. Necroptosis-related genes were significantly downregulated in patients with OC aged ≥ 60 years according to OC transcriptomic data in the TCGA-OV dataset, suggesting that necroptosis participates in the pathophysiology of OC [171]. Thus, it must be considered that necroptosis susceptibility may rely on the cell type-specific unique expression patterns of molecular components involved in the necroptosis pathway. Many current studies are directed at finding the necroptosis-related gene signatures, including the necroptosis-related lncRNA signatures, for predicting the prognosis of OC and differentiating between the “hot tumors” (“T cell inflamed” phenotype) and “cold tumors” (“non-inflamed” phenotype) and predicating the sensitivity of OC cells to various small-molecule inhibitors, immune checkpoint inhibitors, and PBC drugs, which may introduce a novel strategy for OC patients’ treatment [172, 173].

According to the aforementioned data, necroptosis can suppress the initiation and progression of cancer and facilitate cancer therapy; however, numerous studies have revealed that necroptosis can also promote cancer progression, suggesting that necroptosis might play complex roles in carcinogenesis [174, 175]. The released intracellular content can stimulate a variety of cells (including macrophages, fibroblasts, and endothelial cells) to participate in the intrinsic immune response and exacerbate the inflammatory response by releasing inflammatory cytokines, and may generate an immunosuppressive tumor microenvironment. Tissue expression and fluid level of TNF-α have been significantly associated with OC-related inflammatory conditions [176, 177]. High tissue expression of TNF-α targeted receptors, TNFR1 and TNFR2, is correlated with the highest risk of EOC progression [178]. Thus, drugs targeting necroptosis that exhibit potential antitumor efficacy require validation for their clinical feasibility.

### Pyroptosis

Pyroptosis is also a lytic form of RCD but is characterized by the release of pro-inflammatory factors and most frequently occurs upon infection with intracellular pathogens. Rapid lytic cell death was first described by Zychlinsky et al. in infected macrophages after bacterial pathogen (*Salmonella flexneri*) invasion [179]; however, only in 2001 was the term ‘pyroptosis’ coined by Brennan and Cookson to reflect the inflammatory nature of this type of death [180]. These authors were the first to describe that cell death induced by *Salmonella typhimurium* is distinct and independent from the well-known apoptotic caspase-3 activation and that *Salmonella*-infected macrophages are killed by an unusual caspase-1-dependent mechanism of necrosis [181]. Pyroptosis differs from apoptosis and autophagy in cell morphology and is featured by an influx of sodium with water, swelling of cells, and osmotic cellular lysis. The released immunogenic cellular content, which includes damage-associated molecular patterns (DAMPs) and inflammatory cytokines, such as interleukin-1β (IL-1β and IL-18, can trigger local inflammation. Pyroptosis mostly occurs in innate immune system cells such as monocytes, macrophages, and dendritic cells in response to pathogen-associated molecular patterns (PAMPs) expressed on pathogens or cell-derived DAMPs, but recent studies have reported that it can also occur in tumor cells [182].

**Fig. 6:**
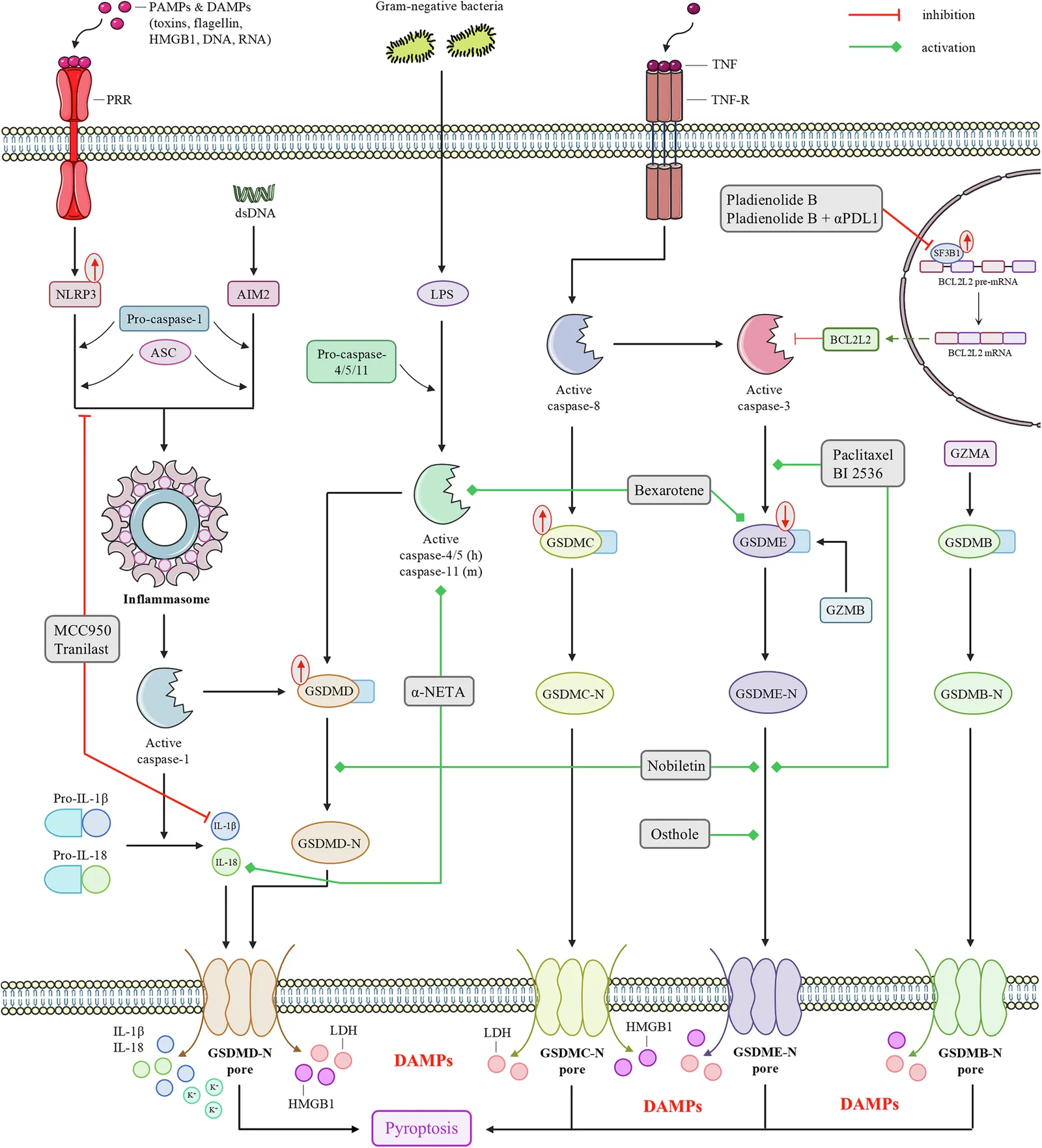
Effect of potential drugs on the pyroptosis pathway. Image provided by Servier Medical Art, licensed under CC BY 4.0.

Pyroptosis, like many other types of RCD, is triggered by activation of the high-molecular-weight complex (inflammasome) and mediated by gasdermin family pore-forming proteins (Fig. 6). The inflammasome is a cytoplasmic oligomeric complex composed of pattern recognition receptors (PRRs), the adapter ASC protein (apoptosis-associated speck-like protein containing a CARD), and the effector caspase-1. The main PRRs in inflammasomes include members of the Nod-like receptor family (NLRPs: NLRP1, NLRP3, and NLRC4) or the PYHIN family member AIM2. Inflammasome activation is defined by oligomerization of the N-terminal PYD domain of NLRPs with the PYD domain of the protein ASC, and ASC itself then recruits pro-caspase-1 via CARD-CARD interactions in response to infectious agents and physiological aberrations [183, 184]. Upon self-cleavage of procaspase-1, active caspase-1 within inflammasomes causes the pro-IL-1β and pro-IL-18 processing into mature forms and specific cleavage of gasdermin D (GSDM-D), leading to the appearance of two domains—GSDM-C (C-terminal domain) and GSDM-N (N-terminal domain). The latter translocates to the cell membrane and binds to acidic phospholipids (such as phosphoinositides) on the inner leaflet of the plasma membrane, causing pore formation. Caspase-4/-5 in humans and caspase-11 in mice, besides caspase-1, also belong to the pro-inflammatory caspase subfamily. The highly homologous sequences between caspase-1 and caspase-11 (caspase-4/-5) imply their functional similarities, but the heterogeneity may be responsible for their mediation of two pathways. Caspase-11 (caspase-4/-5) activates in response to lipopolysaccharide (LPS) on the surface of Gram-negative bacteria. LPS then binds to the CARD domain of procaspase-11, initiates its oligomerization, and forms mature/active caspase-11. This protease cleaves GSDM-D, promoting pyroptosis. It can also trigger NLRP3- and ASC-dependent activation of caspase-1, resulting in the proteolytic maturation of IL-1β [185]. It has been found that not only murine caspase-11, but also human caspase-4/-5, might sense LPS directly [186]. GSDM-D was discovered as the main executioner of pyroptosis among gasdermin family members (GSDM A, B, C, D, E, and DFNB59) [187], while several other gasdermins have been able to form pores that cause pyroptosis. Cisplatin and paclitaxel, as well as extracellular ATP (DAMP), can induce alternative pyroptosis by the caspase-3/GSDM-E pathway instead of the caspase-1/GSDM-D pathway and inhibit the proliferation of lung tumor cells with a high expression level of GSDM-E [188, 189]. Cleavage of GSDM-B by granzyme A secretion from cytotoxic T cells and NK cells induces pyroptosis in a caspase-independent manner [190]. Deletion of caspase-8 blocked GSDM-C cleavage in TNFα-induced pyroptosis in tumor cells [191].

The ability of inflammatory caspases to induce pyroptosis under certain circumstances, when apoptosis and necroptosis are compromised, can be regarded as a possible cancer therapy. Accumulated data show that different chemical substances can evoke the death of OC cells with pyroptotic features. For example, plant-derived molecules, osthole or nobiletin, might stimulate ROS production, decrease MMP (mitochondrial membrane potential), and induce LC3-mediated autophagy and GSDM-E and GSDM-E/GSDM-D-dependent pyroptosis in A2780 and OVCAR3 cells, respectively [192, 193]. The anti-cancer agent, bexarotene, selectively activates caspase-4 and GSDM-E, demonstrating that cell death might be pyroptosis in the ovarian CCC cell line (ES2) [194]. EOC death through pyroptosis with a dramatically augmented level of GSDM-D, caspase-4, LC3B, and IL-18 secretion may be activated by α-NETA treatment [195]. BI 2536, a potent and selective inhibitor of polo-like kinase 1, induced pyroptosis in A2780 OC cells through the caspase-3/GSDM-E pathway [196]. Paclitaxel, as a first-line chemotherapeutic compound in the treatment of OC, can also induce pyroptosis through the caspase-3/GSDM-E pathway in A2780 OC cells [197].

Nevertheless, excessive triggering of pyroptosis in normal cells may result in the creation of chronic inflammation in the surrounding cellular microenvironment and can be a risk factor for the conversion of normal cells to cancer cells due to an increased level of pro-inflammatory cytokines [198]. Engagement of immune system cells, such as M1 macrophages, natural killer cells, neutrophils, and CD8 + T cells in maintenance of chronic pyroptosis-induced inflammation and immune suppressor cells such as MDSCs, M2 macrophages, and Treg cells in suppression of immune attack against tumor cells, makes it very difficult to distinguish the mechanism that regulates promotion or suppression of pyroptosis-driven tumorigenesis. The underlying mechanisms of how pyroptosis could promote the development of certain types of tumors still need to be determined. Increased levels of inflammatory mediators, gasdermins (GSDM-D and GSDM-C), are associated with poor prognosis and shorter survival in EOC patients [199]. Pyroptosis in tumor cells with high gasdermin expression levels can initiate long-lasting upregulation of IL-1β and IL-18, thereby enhancing invasiveness, metastasis, and growth of cancer cells and affecting tumor biological behavior. Hence, most tumor cells inhibit GSDM-E methylation with a DNA methyltransferase inhibitor, suggesting that GSDM-E functions as a tumor suppressor. Activation of GSDM-E suppresses tumor growth by activating anti-tumor immunity through the enhancement of the phagocytosis of tumor cells by tumor-associated macrophages and of the number and functions of tumor-infiltrating NK cells and CD8 + T lymphocytes.

Recent studies have shown that pyroptosis has an impact on tumor proliferation and invasion, which is controlled or regulated by various ncRNAs, but the direct interaction between ncRNAs and pyroptosis progression is poorly understood. Only a few ncRNA molecules have an association with pyroptosis progression, including lncRNA HOTTIP [200] and miR-155 [201] in OC cells. The expression of HOTTIP was significantly upregulated in OC cells and tissues, and its silencing led to the inhibition of cell proliferation and NLRP1 inflammasome-mediated pyroptosis. Importantly, the correlation between predictive pyroptosis-related genes and the clinical stage of OC has been established [202]. Nevertheless, additional experiments are required to determine which exactly ncRNA can modulate pyroptosis in OC cells.

Currently, several groups are focusing on the search for compounds or molecules that can activate the inflammasome and induce pyroptosis; however, the differences in the expression and activity of pyroptosis executors must be taken into consideration since only controlled induction of pyroptosis can improve anti-tumor immune defense and prevent tumor growth to overcome the risk of toxicity of excessive inflammation. Understanding the precise mechanisms of pyroptosis in OC cells and the tumor microenvironment will allow an accurate switch between the different types of cell death to sensitize cancer cells in case of drug chemoresistance or imbalance of gene expression of major players of apoptosis, autophagy, ferroptosis, and necrosis.

## Treatment modalities

Despite the successful response rate of OC to first-line PBC and the development of novel targeted agents, as well as the use of a combinatory approach with conventional therapy (antiangiogenic agents—bevacizumab; PARP inhibitors for BRCA-mutated EOCs—olaparib, rucaparib, and niraparib; immune checkpoint blockade—PD-1/PD-L1 inhibitors, CTLA-4 inhibitors), current treatments still often fail due to the high rate of chemotherapy resistance and the low susceptibility of cancer cells [203].

The ability of OC cells to detect platinum-induced DNA damage for repair is determined by the cancer cells’ ability to resist apoptosis, which in turn affects their susceptibility to chemotherapeutic agents. In response to chemotherapeutic DNA-damaging drugs, complex interactions between various DNA damage-related sensors, activators, repair pathways, and protein complexes happen in cells. It was hypothesized that these participants could be effectively targeted to increase therapy efficacy at the early stage of therapy. Still, later on, cancer cells may activate specific DNA damage repair machinery, which is involved in the development of therapy resistance. Thus, comprehensive research is needed to fully appreciate the roles of DNA damage, response, repair, and their regulation in cancer development, treatment, and chemoresistance [204]. It was also suggested that chemoresistance to PBC is positively correlated with expression of the mutant *TP53* [205]. The cell cycle arrest and induction of apoptosis are the most important functions of p53. In type II EOC, *TP53* is the most mutated and can lose function or gain oncogenic activity. Both gain-of-function (oncogenic) and loss-of-function (loss of p53 activity) mutations occur in HGSOC patients [206]. Missense mutations (R175, Y220, G245, R248, R249, R273, or R282) and hotspot mutations (R273, R248, R175, Y220, I195, C176, G245, S241, Y163) in the DNA-binding domain result in the abolition of DNA-binding functions of p53 (directly or indirectly) and affect apoptotic regulation in HGSOC patients [207]. Among various classes of mutations, loss-of-function mutations in *TP53* are associated with chemoresistance and poorer survival in advanced serous OC [208]. Reactivation of mutant p53 is a promising therapeutic approach, and the APR-246 (PRIMA-1Met) compound has been reported to exert this effect and has already been incorporated into several clinical trials [209]. APR-246 induces expression of the same gene set in mouse lymphoma cells with different statuses of *TP53* (wild-type and knockout). It can eliminate cancer cells through induction of various PCD pathways (including apoptosis, necroptosis, and ferroptosis) depending on the type of cancer cell, proposing that the clinical testing of APR-246 should also be expanded to cancers that express wild-type *TP53* [210]. In wild-type *TP53* cancer cells, p53 becomes upregulated in response to DNA damage, but E3 ubiquitin ligases MDM2 and MDM4 might inhibit p53 stability and activation; thus, the developing and combined use of MDM2 and MDM4 antagonists might result in more effective anti-cancer therapy for non-mutated p53 type I EOC [211]. Antagonist MDM2 (RG7112) may have a potential therapeutic effect on CCC as it significantly reduced the tumor volume of xenografted RMG-I CCC cells [212]. p53 functions as a transcription factor that targets genes to induce cell cycle arrest and apoptosis. It was an attempt to apply p53 single-gene therapy in combination with standard chemotherapy as first-line treatment of OC patients in an international randomized phase II/III trial, which unfortunately did not show adequate therapeutic benefit [213]. Among the possible reasons for the failure of p53 gene therapy, the overexpression of splice variants of p63 and p73 in OC was mentioned. The roles of p63 and p73 in OC pathogenesis and response to therapy are largely unknown, but high p73 protein expression was linked to high-grade, advanced-stage disease and shorter PFS in patients with EOC, and p73 can be exploited for targeted OC therapy and as a predictor of platinum resistance [214]. Among genes that p53 upregulates are the pro-apoptotic genes, including members of the BCL-2 family—*Bax*, *Noxa*, and *Puma* [215], as well as downregulation of the anti-apoptotic gene *survivin*, which inhibits caspase activation via involvement in cell cycle regulation [216]. It has been shown in panels of human OC cell lines, sensitive and resistant to cisplatin and analogs, that a high BCL-2 level supports the sensitivity of cells to platinum drugs and not their resistance [41]. The survival of patients with advanced stages (III-IV) of serous or endometrioid adenocarcinomas is significantly better with BCL-2-negative than with BCL-2-positive tumors [39]. Expression of BCL-2 protein family members is controlled at the transcriptional and translational levels, and their balance determines whether a cell will undergo apoptosis or survive. Thus, BCL-2 family members and their regulators are attractive targets for anti-cancer therapy. Compounds blocking the pro-survival BCL2 family, which mimic the function of BH3-only proteins, have been developed in the last few decades [33]. BH3-mimetics are small molecules that have been validated (but some of them have been discontinued due to severe cytotoxic effects, interaction with non-specific proteins, or low bioavailability) and entered clinical trials and are currently under investigation (ABT-737, ABT-263, APG-1252) as single agents and/or in combination with cytotoxic and targeted agents in solid tumors. Venetoclax (ABT-199), a specific BCL-2 inhibitor, was approved by the FDA in 2016 and successfully applied for a subset of hematological malignancies. Healthy tissues also depend on the pro-survival protein BCL-2, so the notable issue is its on-target effect; however, the first-in-human study has indicated good tolerability of Pelcitoclax (APG-1252) in OC and in other advanced solid tumors [217]. The ability of WEHI-539, a selective BCL-XL inhibitor, to potentiate the activity of carboplatin in six OC cell lines has been investigated, while BCL-XL appears to be more frequently overexpressed in solid tumors, including OC. WEHI-539 and also ABT-737 (binding BCL-2, BCL-XL, and BCL-W proteins), but not ABT-199, were synergistic with carboplatin in cell growth assays and potentiated cell death in tested OC cell lines [218]. It has been shown that in HGSOC patients, ABT-737 induced apoptosis as a single agent. However, its efficacy was not improved by the addition of carboplatin, and the absence of response to ABT-737 was correlated with the absence or low expression of BIM [219]. Monotherapy in patients with platinum-resistant HGSOC had poor activity that was not correlated with the expression of BIM, MCL-1, and P-ERK, and thrombocytopenia was the major side effect [220]. OC is usually less sensitive to BH3-mimetics due to dependence on high expression of BCL-XL, compared to hematological malignancies, and the tolerability of treatment with several BH3-mimetic compounds needs to be determined. In view of the fact that cancer cells rely on two or even more pro-survival BCL-2 proteins to escape death in response to cytotoxic drugs, the effective killing of cancer cells may require two or more BH3-mimetics or a combination with different anti-cancer agents. Since the application of BH3 mimetics is associated with significant thrombocytopenia, additional strategies would be necessary to overcome this adverse effect. Given that pro-apoptotic genes *Puma* and *Noxa* are direct transcriptional targets of *TP53*, it was proposed that targeting these proteins can effectively kill the cancer cells. Accordingly, in PUMA adenovirus-infected A2780 OC cells, elevated PUMA induces ROS generation, DNA damage response, and JNK activation, which contribute to apoptosis [221]. Overexpression of *Noxa* in OC cells and treatment with gemcitabine showed a more significant cytotoxic effect against OC cells, and it could be potentially useful as a chemosensitizer in OC therapy [222]. While cancer cells have different expression codes among BCL-2 family proteins and different responses to BH3 mimetics, it becomes more challenging to identify the right agents for patients to reverse chemoresistance or induce death in OC cells. In addition, the BH3 profiling technique and the dynamics of BH3 profiling have been suggested. This can be used to predict the susceptibility of OC to antitumor agents and select the most effective drugs [32].

In OC, as in many other cancer cells, overexpression of survivin has been demonstrated [223], and its expression was shown to correlate with poor prognostic markers (high grade, histologic type, and p53 mutation) in most OCs [224]. Downregulation or inhibition of survivin results in spontaneous apoptosis or sensitization of OC cells to chemotherapy and radiotherapy [225]. There are several survivin inhibitors under clinical evaluation, but some of them exhibit very limited antitumor efficacy in phase I/II clinical trials, possibly due to compensatory upregulation of other IAP family proteins or the induction of different isoforms of survivin. Therefore, the therapeutic strategy has to identify an effective synergistic combination of IAP inhibitors [226]. Possibly it might be the combination that includes c-IAP1/2 inhibitors (ASTX660, birinapant, and DEBIO 1143, all of which are under clinical trials), XIAP inhibitors (SM-164 and LCL161), and survivin inhibitors [227]. While the expression of survivin is minimal in normal tissues and high in almost all types of malignancies, it makes survivin an attractive target for various cancers, including T-cell-based immunotherapy of OC. DPX-Survivac is a novel T cell-activating therapy compound containing a mix of HLA class I peptides designed to evoke a T cell response against survivin. DPX-Survivac was shown to stimulate a sustained T cell survivin-specific response and increase infiltration of T cells into tumors [228]. Nevertheless, the identification of suitable tumor-associated antigens (TAAs) as targets for immunotherapy in OC is challenging. The usage of a systematic vetting algorithm has identified KIF20a, CT45, and LY6K to be highly suitable for targeted T-cell therapy in OC [229]. Thus, the KIF20A-based thermosensitive hydrogel vaccine (K/RLip@Gel) demonstrated the synergistic improvement of PD-L1 blockade and therapy outcomes in subcutaneous and orthotopic cell-derived xenograft models, along with immune-humanized patient-derived xenograft hepatocellular carcinoma models [230]. Consequently, all these antigens need to be tested for employment in T cell-based immunotherapy of OC treatment. p53 may also act as an antigen for a specific anti-tumor immune response. Unfortunately, current p53 vaccines do not improve patients’ survival in different phase trials [details in 231].

However, developing oncolytic virotherapy as a novel antitumor modality in combination with chemotherapy, radiotherapy, and immunotherapy has therapeutic potential. The benefit of oncolytic virus therapy, which causes direct lysis of cancer cells and subsequent release of tumor antigens that induce tumor-specific immunity and immunogenic cell death, has been demonstrated in several preclinical studies [232]. Advanced metastatic EOCs are often immunosuppressive and therefore challenging to treat with conventional immunotherapy, and the combination of oncolytic viruses and antigen-specific immunotherapy can lead to enhanced anti-tumor effects against ovarian tumors.

The action of some anti-cancer agents on tumor cells results in activation of not only apoptotic but also non-apoptotic cell death modalities. For example, DNA damage demands the substrates for DNA repair, which are provided by autophagy recycling. Wild-type p53 can activate autophagy genes (*Dram*, *Ulk1*, and *Atg7*), and in turn, activation of autophagy can suppress p53 and promote resistance to DNA-damaging agents [233]. Mutant p53 may repress expression of several autophagy-related proteins (Beclin-1, DRAM, ATG12, and SESN1/2), which results in dysregulation of autophagy [98]. It has been proposed that in some cancer-associated *TP53* mutants, chemosensitivity to DNA damage will be restored, but further studies have shown that the subcellular localization of p53 can also mediate functional responses between apoptosis and pro-survival autophagy. The aberrant up-regulation of autophagy can lead to cell death, and this feature can be exploited for cancer therapy, but with consideration of its complex role as a tumor promoter in the initial stage of tumorigenesis. The level of autophagy in the cancer cell is greatly affected by vascularization, inflammation, and stromal cell infiltration, and small ncRNAs have an impact on autophagy as important epigenetic modulators [234]. The most beneficial effects may be achieved if autophagy enhancers or autophagy inhibitors are applied along with anti-neoplastic therapies in accordance with the actual level of ongoing autophagy in the tumor cells, which is mediated by genetic mutations, the epigenetic regulation of relevant genes, and the tumor microenvironment. Several highly specific autophagy inhibitors have been developed that inhibit the interaction of autophagy-related proteins, including SUPR4B1W, which targets the ATG4B-LC3 protein-protein interaction. SUPR4B1W demonstrated good results in sensitization of OC cells to cisplatin [235] and potentially can be used as cell-penetrating macrocyclic peptides instead of chloroquine and hydroxychloroquine, two autophagy inhibitors in clinical trials, which suffer from poor pharmacokinetics and high toxicity at therapeutic dosages.

Platinum resistance of OC is associated with elevated intracellular GSH concentration [236]. GSH serves as a critical regulator of ferroptosis. Modulation of ferroptosis susceptibility of cancer cells through GSH metabolism uncovers its potential as a therapeutic target [237]. It has been proposed that a modest reduction in GSH levels within cancer cells may enhance their susceptibility to oxidative stress and chemotherapy. The level of GSH can be affected by the enzymatic system, which participates in its synthesis and targets transport membrane proteins (SLC7A11, MRP1). The therapeutic targeting of mutant p53 protein suppresses the expression of SLC7A11, triggering ferroptosis [238]. The development of pharmaceutical agents targeting GSH metabolism presents significant importance in the precise regulation of GSH concentration and is currently an unsolved task and a goal for future personalized medicine.

Diverse DNA-damaging agents culminate in the induction of PCD via activation of not only apoptosis but also necroptosis, which is mediated by the RIPK1-RIPK3-MLKL pathway. Necroptosis appears to be suppressed in numerous tumor cell lines due to loss of RIPK3 expression [239]. A decreased level of RIPK3 correlates with resistance to necroptosis, and BRAF and AXL oncogene overactivation can act as the driving forces for the loss of RIPK3 in progressive stages of OC. Understanding the mechanism of escape from necroptosis in OC may lead to a way to restore necroptosis sensitivity. One of the possibilities is to inhibit oncogenes BRAF and AXL to rescue the loss of RIPK3 expression and regain necroptosis. The AXL inhibitor (AXLi) BGB324 alone or in combination with the clinically relevant BRAF inhibitor (BRAFi) vemurafenib has already been tested on melanoma cells and showed promising results in the stimulation of ferroptosis and affecting other cell death mechanisms [240].

As mentioned above, the potential for triggering pyroptosis is also an interesting targeting therapy for apoptotic drug-resistant cancers, but the inflammatory substances (IL-1β, IL-18, and alarmins) released during pyroptosis may create a persistent inflammatory milieu. Excessive or uncontrolled pyroptosis has been proven to induce chronic inflammation, and the mechanisms of regulation of pyroptotic pathways in the complex environment that surrounds and interacts with cancer cells (tumor microenvironment) and affects tumor behavior demand detailed exploration for minimizing adverse side effects [241]. Chronic inflammation is a critical driver of cancer progression, contributing to immune evasion, tumor growth, and metastasis in OC [242]. Potential therapeutic substances targeting pyroptotic pathway participants are under development, including selectively inflammasome-targeting agents. OC showed increased expression of *NLRP3*, indicating worse OS [243]. Evaluation of the effect of MCC950, a specific inhibitor of the NLRP3 inflammasome, on cell survival and IL-1β production showed reduced survival and inhibition of IL-1β secretion in primary human endometrial stromal cells [244]. However, MCC950 has not been used in a clinical trial because it has potential liver toxicity [245]. A high-throughput screen of an FDA/EMA-approved drug library identified that another clinically applied drug, tranilast, which directly targets NLRP3 [246], could reduce cisplatin resistance. Tranilast has been tested in platinum-resistant OC cells, and the results indicated that it promoted cisplatin toxicity [247]. A proinflammatory environment may promote carcinogenesis through not only the sustained activation of inflammatory pathways but also through the immune cell infiltration into the tumor microenvironment [243]. The low cytotoxic immune cell infiltration in OC is related to the overexpression of SF3B1. Inhibition of SF3B1 by pladienolide B induces pyroptosis in OC cells and polarization of macrophages and infiltration of cytotoxic lymphocytes in mouse allograft tumors. Moreover, the application of an SF3B1 inhibitor together with αPDL1 increased the population of CD8+ and IFN-γ + CD8 + T cells more significantly in combination and offers preclinical evidence of excellent efficacy of synergizing immune checkpoint blockade immunotherapy with pyroptosis-induced improvement of the immune microenvironment during OC treatment [248].

## Conclusion

Ovaries play a crucial role in women’s fertility, and, despite the advancements in cancer therapies, the number of patients who face drug resistance is still high, and late complications affecting fertility and hormonal balance may arise [249]. The review of phase I trials in OC, published between 2012 and 2023, with different treatment strategies (chemotherapy-only, chemotherapy plus non-chemotherapy agents, and chemotherapy-free) and outcomes showed that chemotherapy-free strategies display a more favorable safety profile, while regimens combining chemotherapy and non-chemotherapy agents seem to be more effective compared to chemotherapy-only approaches [250]. Currently, a limited number of therapies have gained approval, including bevacizumab, PARP inhibitors, mirvetuximab soravtansine, and trametinib for low-grade serous OC beyond chemotherapy. Different OC subtypes require therapeutic approaches that enhance the efficacy of treatment. Identification of biomarkers such as *BRCA* mutation status and HRD to predict the response to PARP inhibitors does not fully reflect the complexity of OC, because HRD status does not consistently predict PARP inhibitor sensitivity [251]. PD-1/PD-L1 blockade has shown limited success in OC trials, and a combination of PD-1/PD-L1 blockade and chemotherapy or PARPi failed to gain sufficient survival in clinical trials [252]. Such unsuccess may be due to a poor understanding of the mechanisms that regulate the temporal evolution of malignant and myeloid cell networks with disease progression. Work of Ghisoni et al. revealed BRCA-dependent TIL: myeloid crosstalk as key to persistent immunogenicity in recurrent OC and provided new targets for differential treatment approaches according to BRCA/HRP status [253].

Understanding how OC cells can acquire the capability to sustain proliferative signaling and resist cell death will contribute to effective targeted therapy with the most likely positive outcome. Progress in identifying apoptosis’s extrinsic and intrinsic pathways has led to the identification of targets for anti-cancer therapy. However, cancer cells frequently acquire resistance to apoptosis through the overexpression of anti-apoptotic proteins, thereby promoting tumorigenesis and sustained malignant progression. In response to these limitations, recent advances in targeted therapies have increasingly focused on alternative, non-apoptotic forms of cell death, including autophagy, ferroptosis, necroptosis, pyroptosis, and others. These approaches elucidate distinct signaling pathways and molecular mechanisms beyond apoptosis, offering potential therapeutic targets and significant promise for tumor prevention and treatment [254–256]. Supplementary Table 1 summarizes currently available therapeutic strategies targeting distinct PCD pathways and highlights the main barriers to clinical application.

As described above, increased or decreased expression of genes regulating different types of cell death is associated with a good or poor prognosis for patients. Currently, the treatment of patients with high-specificity inducers of certain PCD alone is not likely an effective strategy, because OC needs combination treatment with chemo- or immunotherapy. It is known that chemotherapeutic agents induce different types of PCD. In this context, the search for signatures of genes responsible for different PCD and their correlation with the patients’ clinical characteristics would help to create prognostic models of the patient’s response to treatment. Moreover, an integration of molecular and cellular profiling of patients will enable them to select those who would benefit from a coordinated and structured approach to achieve more effective treatment and better success in combating OC. Spatial proteo-transcriptomic profiling may be such an approach, as it already helps to validate the combination of milciclib (targets CDK4/6) and mirvetuximab (targets FOLR1) to achieve significant LGSC tumor reduction in vivo [257]. Thus, despite the significant advances in OC therapy in recent years, the efforts of specialists in various fields are required to gain new knowledge and develop new drugs that target various types of cell death for the successful treatment of OC patients. There is no doubt that these attempts will be successful.

## Supplementary information

Supplementary Table
