# Supplementary material for: Cell death-based treatment of ovarian cancer
Source: Cell Death Dis. 2026 Aug 3;17(1):923. doi: 10.1038/s41419-026-09133-x (PMC13720068; doi:10.1038/s41419-026-09133-x)
Supplement: Supplementary file 1 [file 41419_2026_9133_MOESM1_ESM.docx]

**Supplementary Table 1**. PCD-targeted therapeutic strategies and their translation limitations.

| **Type of PCD** | **Target molecules/**  **pathways** | **Therapeutic agents** | **Model characteristics** | **Level of evidence** | **Reference** | **Major translational limitations** |
| --- | --- | --- | --- | --- | --- | --- |
| Apoptosis (intrinsic) | BCL-2 | ABT-199 + carboplatin | OVCAR-3, OVCAR-4, OVCAR-8, OVSAHO, COV-362 (HGSOC); IGROV1 (ovarian endometrioid adenocarcinoma) | **Preclinical trials**: *in vitro* cell culture | 219 | 1) Targeted BCL-2 inhibitors (like ABT-199, which has demonstrated success in hematological malignancies) have minimal effect on OC cells even in preclinical studies, because OC rely more on MCL-1 and BCL-XL to avoid death.  2) Inhibition of BCL-XL (e.g., with ABT-263) is associated with the development of thrombocytopenia. Strategies to overcome thrombocytopenia are needed (e.g., novel dual BCL-2/BCL-XL inhibitor APG-1252 is currently in clinical trial – NCT05691504; estimated completion data: 2027).  3) Long-term inhibition of MCL-1 can increase the risk of cardiotoxicity and disrupt hepatocellular homeostasis.  Further studies of MCL-1 inhibitors in the context of OC treatment, as well as the use of targeted delivery to minimize systemic toxicity, may help overcome current limitations.  4) Single-agent BH3-mimetics typically have limited efficacy (e.g., ABT-263 monotherapy) because OC cells often overexpress several pro-survival proteins of the BCL-2 family. Two or more BH3-mimetics or a combination with different anti-cancer agents are required. |
| Apoptosis (intrinsic) | BCL-2, BCL-XL, BCL-W | ABT-737 | OVCAR-3, OVCAR-4, OVCAR-8, OVSAHO, IGROV1, COV-362 | **Preclinical trials**: *in vitro* cell culture | 219 |  |
| Apoptosis (intrinsic) | BCL-2, BCL-XL, BCL-W | ABT-737 ABT-737 + carboplatin | Tumor nodes from patients with HGSOC who were chemo-naïve and had undergone surgery, N = 25 | **Preclinical trials:** *ex vivo* tumor nodes from patients | 220 |  |
| Apoptosis (intrinsic) | BCL-2, BCL-XL, BCL-W | ABT-263 | Patients with platinum-resistant HGSOC who were heavily pre-treated,  N = 46 | **Сlinical trials:**  Phase II (PFS, efficacy) | 221 |  |
| Apoptosis (intrinsic) | BCL-2, BCL-XL | APG-1252 | Patients with metastatic solid tumors (N = 50), including OC (N = 5) | **Сlinical trials:**  Phase I (safety, dosage, PD, PK) | 218 |  |
| Apoptosis (intrinsic) | BCL-2, BCL-XL | APG-1252 + cobimetinib | Patients with recurrent EOC (N = 42) | **Сlinical trials:**  Phase I in progress (safety, tolerability, PD, PK, dosage) | [NCT05691504](https://clinicaltrials.gov/study/NCT05691504) |  |
| Apoptosis (intrinsic) | BCL-XL | WEHI-539 | OVCAR-3, OVCAR-4, OVCAR-8, OVSAHO, IGROV1, COV-362 | **Preclinical trials**: *in vitro* cell culture | 219 |  |
| Apoptosis (intrinsic) | BCL-XL, MCL-1 | siRNA BCL-XL + siRNA MCL-1 + cisplatin | Chemoresistant EOC cells SKOV3 (high-grade serous cystadenocarcinoma, ascites), IGROV1-R10 | **Preclinical trials**: *in vitro* cell culture | 55 |  |
| Apoptosis (intrinsic) | BCL-XL/BCL-2, MCL-1 | ABT-737 + cisplatin/carboplatin ABT-737 + siRNA MCL-1 | Chemoresistant EOC cell lines SKOV3 and IGROV1-R10 | **Preclinical trials:**  - *in vitro* cell culture  - *ex vivo* tumor nodes from CDX | 56 |  |
| Apoptosis (intrinsic) | BCL-XL, BCL-2, MCL-1 | Metformin Metformin + cisplatin | HGSOC cell lines OVCAR-3, OVCAR-4 | **Preclinical trials**: *in vitro* cell culture | 57 |  |
| Apoptosis (intrinsic) | BCL-XL, BCL-2, MCL-1 (↓);  BAX, pBAD (↑) | Metformin + carboplatin/paclitaxel | Patients with advanced EOC (FIGO III-IV),  N = 15 | **Сlinical trials:**  Phase I (safety, tolerability, dosage) | 58 |  |
| Apoptosis (intrinsic) | p53 | APR-246 + carboplatin/pegylated liposomal doxorubicin hydrochloride (PLD) | Patients with platinum-sensitive recurrent HGSOC with mutated p53 (Phase Ib: N = 36, Phase II: N = 211) | **Сlinical trials:**  Phase Ib/II (dose-limiting toxicities, safety, PFS, overall response rate) | [NCT02098343](https://clinicaltrials.gov/study/NCT02098343) | 1) Lack of clinical benefit of APR-246 in patients with recurrent HGSOC with p53 mutations.  Mutation-specific targeting (multiple TP53 mutations leading to functionally distinct mutant protein structures), including in combination with other targeted anticancer drugs and immunotherapy, is likely to achieve better results.  2) p53-based gene therapy has not demonstrated an appropriate therapeutic effect in patients with OC for the following reasons: - multiple genetic alterations in OC, making single-gene replacement ineffective; - dominant negative interplay between wild-type p53 and dominant p53 mutants and splice variants of p63 and p73 which are overexpressed in OC; - limitations of the delivery system (adenoviral vector system and intraperitoneal delivery method); - adenovirus-neutralizing antibodies in ascitic fluid of OC patients.  3) Treatment with MDM-2 inhibitors in clinical practice leads to various problems, including acquired resistance, co-overexpression of MDMX, hematological and gastrointestinal toxicity.  Combination therapies involving chemo- and immunotherapy, dual MDM-2/MDMX inhibitors, and MDM-2 degraders (proteolysis-targeting chimeras) may enhance the efficacy of MDM2 inhibitors. |
| Apoptosis (intrinsic) | p53 | *TP53* gene-therapy (adenoviral vectors carrying wild-type *TP53) +* carboplatin or paclitaxel) | Patients with primary FIGO III OC with *TP53* mutations who had undergone surgical cytoreduction | **Сlinical trials:**  Phase II/III randomised international trial (terminated after first interim analysis) | 214 |  |
| Apoptosis (intrinsic) | MDM-2 | RG7112 | *TP53* wild-type CCC cell lines OVISE,  RMG-I, JHOC-7, OVTOKO | **Preclinical trials**:  - *in vitro* cell culture - *in vivo* CDX (RMG-I, OVISE) | 213 |  |
| Apoptosis (intrinsic) | PUMA | Recombinant adenovirus expressing *Puma* (Ad-PUMA) | *TP53* wild-type A2780s (chemosensitive), *TP53* null SKOV3 (chemoresistant) | **Preclinical trials**:  - *in vitro* cell culture | 222 | Therapy targeting NOXA and PUMA in OC has only been evaluated in preclinical studies and requires further clinical trials; however, currently, potential limitations of this approach comprise the risk of insufficient tumor specificity and off-target toxicity, the need for safe and effective gene delivery without affecting healthy tissues, etc. |
| Apoptosis (intrinsic) | NOXA | pcDNA vector encoding *Noxa* (pcDNA3.1-Noxa) + gemcitabine | A2780, COC1 (poorly differentiated OC; ascites) | **Preclinical trials**:  - *in vitro* cell culture - *in vivo* CDX | 223 |  |
| Apoptosis | XIAP | siRNA XIAP + cisplatin | OC cell line KK derived from a patient with CCC | **Preclinical trials**:  - *in vitro* cell culture | 60 | 1) Restricted delivery of IAP-targeted antisense oligonucleotides to tumor cells.  Accordingly, optimization of delivery systems is necessary.  2) Limited efficacy and toxicity of SMAC-mimetics (particularly in monotherapy): - LCL161 + topotecan: increased myelosuppression, no effect after drug combination; birinapant: lack of clinical benefit.  So, the selection of optimal combination therapy regimens is an important goal.  3) Adaptive resistance due to compensatory activation of alternative signaling pathways.  Using combination therapy will bring more benefits. |
| Apoptosis | XIAP | Antisense oligonucleotide against XIAP (AEG35156) | A2780s (cisplatin-sensitive), A2780cp (cisplatin-resistant), ES-2 (poorly differentiated CCC) | **Preclinical trials**:  - *in vitro* cell culture - *in vivo* CDX (A2780cp) | 61 |  |
| Apoptosis | XIAP | Phenoxodiol | Docetaxel-resistant SKOV3, R182 (primary EOC cells isolated from ascites of FIGO III/IV patients) | **Preclinical trials**:  - *in vitro* cell culture | 63 |  |
| Apoptosis | XIAP | LCL161 + Topotecan | Patients with platinum-resistant recurrent EOC (N = 19) | **Сlinical trials:**  Phase Ib (dosage, safety); study was terminated early | [NCT02649673](https://clinicaltrials.gov/study/NCT02649673) |  |
| Apoptosis | Survivin | MX106 | EOC cell lines OVCAR-3, paclitaxel-resistant OVCAR-3, SKOV3 | **Preclinical trials**:  - *in vitro* cell culture - *in vivo* orthotopic CDX (SKOV3) | 62 |  |
| Apoptosis | Survivin | DPX-Survivac DPX-Survivac + intermittent,  low-dose cyclophosphamide | Patients with advanced, recurrent OC after initial chemotherapy and debulking surgery (Phase I: N = 19,  Phase II: N = 22) | **Сlinical trials:**  Phase I (safety, dosage, immunogenicity) Phase II (efficacy, tolerability, immunogenicity) | 229 [NCT01416038](https://clinicaltrials.gov/study/NCT01416038) [NCT02785250](https://clinicaltrials.gov/study/NCT02785250) |  |
| Apoptosis | IAP1/2 | DEBIO 1143 DEBIO 1143 + paclitaxel/carboplatin | Patients with platinum-refractory EOC (N = 20) | **Сlinical trials:**  Phase I (safety, dosage, PK, PD) | [NCT01930292](https://clinicaltrials.gov/study/NCT01930292) |  |
| Apoptosis | IAP1/2 | DEBIO 1143 DEBIO 1143 + paclitaxel/carboplatin | Patients with newly diagnosed advanced EOC before surgery,  N = 35 | **Сlinical trials:** Phase II (study was terminated early) | [EudraCT Identifier 2015-005137-42](https://www.clinicaltrialsregister.eu/ctr-search/search?query=2015-005137-42) |  |
| Apoptosis | IAP1/2 | Birinapant | Patients with relapsed platinum-refractory or platinum-resistant EOC, N = 11 | **Сlinical trials:** Phase II (efficacy, safety, etc.) The study was terminated due to a lack of clinical benefit | [NCT01681368](https://clinicaltrials.gov/study/NCT01681368) |  |
| Apoptosis | IAP1/2 | Birinapant + carboplatin | Patients with recurrent HGSOC, N = 34 | **Сlinical trials:**  Phase I/II (terminated early due to lack of funding) | [NCT02756130](https://clinicaltrials.gov/study/NCT02756130) |  |
| Apoptosis | IAP1/2, XIAP | ASTX660 | Patients with advanced solid tumors, including OC | **Сlinical trials:**  Phase I/II (safety, dosage, efficacy, etc.) | [NCT02503423](https://clinicaltrials.gov/study/NCT02503423) |  |
| Apoptosis (extrinsic) | DR4/5 | recombinant (GST)-TRAIL + cisplatin/paclitaxel | IGROV1, A2780, SKOV3 | **Preclinical trials**:  - *in vitro* cell culture | 73 | 1) Limited anti-tumor activity of rhTRAIL and DR4/5 agonist antibodies in patients.  2) Very short half-life of rhTRAIL and insufficient agonist activity.  3) Overexpression of decoy receptors and binding of rhTRAIL to them.  4) rhTRAIL and bivalent DR4/5 agonistic antibodies fail to effectively induce the high-order receptor clustering, which results in weak apoptotic signals. |
| Apoptosis (extrinsic) | DR4/5 | recombinant (GST)-TRAIL + cisplatin/ paclitaxel/  doxorubicin | 12 chemoresistant EOC cells: SKOV3; 222 (ovarian mixed germ cell tumor; ascites); A224, A364, A547, UCI 101, UCI 107 (ovarian serous adenocarcinoma); A2780/AD10 (doxorubicin-resistant), CAOV-3 (HGSOC); A2780/CP70 (cisplatin-resistant); OVCAR-3, OVCA-429 (ovarian cystadenocarcinoma) | **Preclinical trials**:  - *in vitro* cell culture | 74 |  |
| Apoptosis (extrinsic) | DR4/5 | rhTRAIL + topotecan/ carboplatin/paclitaxel/doxorubicin | ES-2, A2780, CAOV-3, A2780/AD10, MZ-15 and MZ-26 (ovarian serous carcinoma) | **Preclinical trials**:  - *in vitro* cell culture | 75 |  |
| Apoptosis (extrinsic) | TRAIL/caspase-8 | Epothilone B | OV-90 (ovarian papillary serous adenocarcinoma) | **Preclinical trials**:  - *in vitro* cell culture | 77 |  |
| Apoptosis (extrinsic) | DR4/5 c-FLIP (↓) | rhTRAIL + Reovirus | OVCAR-3, SKOV3, PA-1 (ovarian mixed germ cell tumor, ascites) | **Preclinical trials**:  - *in vitro* cell culture | 78 |  |
| Apoptosis (extrinsic) | DR4/5 c-FLIP (↓) | rhTRAIL + Trichostatin A | SKOV3, Hey8 | **Preclinical trials**:  - *in vitro* cell culture | 79 |  |
| Apoptosis (extrinsic) | DR5 | mAb TRA-8 | Patients with advanced OC (FIGO IIIC-IV) undergoing initial cytoreductive surgery (tumor specimens) | **Preclinical trials**: - *ex vivo* model of primary OC (tumor specimens from patients) | 80 |  |
| Apoptosis (extrinsic) | DR4/5 | Dulanermin (rhTRAIL) | Patients with advanced cancer (N = 71), including OC (N = 9) | **Сlinical trials:** Phase I (safety, tolerability, dosage) | 81 |  |
| Apoptosis (extrinsic) | DR4 | Mapatumumab | Patients with malignant solid tumors refractory to conventional therapy (N = 41), including OC (N = 9) | **Сlinical trials:** Phase I (safety, toxicity, dosage, PK) | 82 |  |
| Apoptosis (extrinsic) | DR5 | Tigatuzumab (CS-1008) + paclitaxel/carboplatin | Patients with FIGO IIIC/IV OC after suboptimal debulking surgery and no prior disease therapy (N = 24) | **Сlinical trials:** Phase II (efficiency, overall response, response rate) | [NCT00945191](https://clinicaltrials.gov/study/NCT00945191) |  |
| Apoptosis (intrinsic + extrinsic) | TRAIL, DR5 BCL-2, BCL-XL, BCL-W | ONC201 + ABT-263 | IGROV1, SKOV3, OVCAR-3 | **Preclinical trials**:  - *in vitro* cell culture | 83 | 1) Gene delivery challenges (immunogenicity, off-target effects, adenovirus-neutralizing antibodies in ascitic fluid, etc.). 2) Interactions between gene therapy vectors, host immune response, and tumor microenvironment remain unclear. 3) Potential toxicity and damage to normal cells. |
| Apoptosis (intrinsic + extrinsic) | BAX TRAIL | *Bax* and *Trail* gene therapy (adenoviral vectors) | SKOV3ip (highly invasive and aggressive SKOV3) | **Preclinical trials**:  - *in vitro* cell culture - *in vivo* CDX | 84 |  |
| Apoptosis + Ferroptosis | LIP, ferritin (↑), GSH (↓) ATP, MMP (↓) | Sodium molybdate | SKOV3, A2780 | **Preclinical trials**:  - *in vitro* cell culture - *in vivo* CDX (SKOV3) | 85 | Insufficient clinical validation, risk of systemic toxicity, and negative impact on normal cells. |
| Apoptosis + Ferroptosis | GALNT14 | shRNA GALNT14 | SKOV3, OVCAR-3 | **Preclinical trials**:  - *in vitro* cell culture | 86 |  |
| Autophagy  (pro-survival) | Autophagosome-lysosome fusion (↓) | Chloroquine + carboplatin | Primary OC cells from ascitic effusions of patients with EOC | **Preclinical trials**:  - *in vitro* cell culture - *in vivo* PDX | 105 | 1) The dual role of autophagy in OC: suppressing tumor progression in early stages of OC and its stimulation in later stages. This makes it difficult to predict the therapeutic potential of autophagy suppression or activation and increases the risk of unintentionally accelerating disease progression.  2) The lack of validated biomarkers to select patients who would benefit from autophagy inhibition/stimulation.  3) Limitations of existing agents: Chloroquine and hydroxychloroquine, two autophagy inhibitors in clinical trials, are non-specific (act at a late stage of autophagy) and suffer from poor pharmacokinetics and high toxicity at therapeutic dosages.  4) Ineffective monitoring of autophagy in patients: existing methods provide static measurements of autophagy-related proteins rather than assessing dynamic autophagic flux, which often leads to inaccurate interpretations.  5) The use of nсRNAs to modulate autophagy may be limited by the following potential issues: difficulty of delivery, instability in vivo, multi-target effect and low specificity, and off-target toxicity. |
| Autophagy  (pro-survival) | Autophagosome-lysosome fusion (↓) | Chloroquine + cisplatin | A2780 (cisplatin-sensitive), A2780-CP20 (cisplatin-resistant); OC cells from a patient with platinum-resistant EOC (FIGO IIIC) | **Preclinical trials**:  - *in vitro* cell culture - *in vivo* CDX (A2780-CP20) and PDX | 106 |  |
| Autophagy (death-inducing) + Apoptosis | LC3-II/I (↑) | Metformin | SKOV3 | **Preclinical trials**:  - *in vitro* cell culture | 107 |  |
| Autophagy (pro-survival) + Apoptosis | Autophagosome-lysosome fusion, p62 (↓) ROS, DSBs (↑) HRR (↓) | Chloroquine + Panobinostat | IGROV1, OVCAR-8, SKOV3, A2780 | **Preclinical trials**:  - *in vitro* cell culture | 117 |  |
| Autophagy (pro-survival) + Apoptosis | Autophagosome-lysosome fusion (↓) LC3-II (↓), p62 (↑) BCL-2 (↓), BAX (↑) | Paeonol + autophagy inhibitors (3-methyladenine/ hydroxychloroquine) | SKOV3, A2780 | **Preclinical trials**:  - *in vitro* cell culture - *in vivo* CDX (A2780) | 118 |  |
| Autophagy (pro-survival) + Ferroptosis | LPO, MDA, Fe2+ (↑) ATF4 (↓) GSH metabolism (↓) ATG3/5/12, p62, BECN1 (↓) | Anisomycin | Cancer stem cells from patients with OC (CD44+/CD133+) | **Preclinical trials**:  - *in vitro* cell culture | 108 |  |
| Autophagy (death-inducing) + Necroptosis | p62 (↓), LC3-I (↑) RIPK1/pRIPK1 (↑) Inflammatory cytokines (↑) | VDX-111 | COV504 (HGSOC, pleural effusion), Kuramochi (HGSOC; ascites), OV7 (poorly differentiated OC, mixed tumor type), OVCAR-3, PEO1 and SNU8 (serous ovarian adenocarcinoma; ascites); OC cells from a patient with untreated FIGO IIIC HGSOC | **Preclinical trials**:  - *in vitro* cell culture - *in vivo* PDX | 109 |  |
| Autophagy (death-inducing) + Ferroptosis | LC3-II/I, ATG5, BECN1 (↑), p62 (↓) GPX4, GSH, xCT (↓) Fe2+, MDA (↑) | Harmine | SKOV3 | **Preclinical trials**:  - *in vitro* cell culture - *in vivo* CDX | 110 |  |
| Autophagy + Pyroptosis | NLRP3, caspase-1, IL-18, GSDM-D (↑) Autophagosome-lysosome fusion (↓) | Harmine + Chloroquine | SKOV3 | **Preclinical trials**:  - *in vitro* cell culture - *in vivo* CDX | 110 |  |
| Autophagy (pro-survival) | miR-12515p/TBCC  /α/β-tubulin axis LC3-II/I (↑), p62 (↓) | miR-1251-5p inhibitor | SKOV3, HEY | **Preclinical trials**:  - *in vitro* cell culture - *in vivo* CDX | 111 |  |
| Autophagy (death-inducing) | lncRNA HULC => ATG7 (↑), ITGB1 (↓) => LC3-II/I, LAMP1 (↑), p62 (↓) | siRNA HULC | OVCAR-3, A2780 | **Preclinical trials**:  - *in vitro* cell culture - *in vivo* CDX (A2780) | 112 |  |
| Autophagy  (pro-survival) | lncRNA LOC730101 => pBECN1 (↓) => LC3-II (↓), p62 (↑),  BCL-2 (↓) | Lentivirus-mediated overexpression of LOC730101 + cisplatin/niraparib | OVCAR-3, A2780 | **Preclinical trials**:  - *in vitro* cell culture - *in vivo* CDX (OVCAR3) | 113 |  |
| Autophagy  (pro-survival) | ncRNA circRAB11FIP1 => ATG7/14 (↓) | Lentivirus-mediated silencing of circRAB11FIP1 | SKOV3, A2780 | **Preclinical trials**:  - *in vitro* cell culture - *in vivo* CDX (SKOV3) | 115 |  |
| Autophagy  (pro-survival) | LC3-II/I, ATG4 | SUPR4B1W + cisplatin/carboplatin | HGSOC cell lines OVCAR-5, OVCAR-8, HEY | **Preclinical trials**:  - *in vitro* cell culture - *in vivo* CDX (OVCAR8) | 236 |  |
| Ferroptosis | system Xc- | Erastin | OC tumor-initiating cells ("cancer stem cells") | **Preclinical trials**:  - *in vitro* cell culture - *in vivo* CDX | 131 | 1) Limited clinical efficacy of FINs: poor pharmacokinetics, insufficient tumor specificity, and consequential toxicity in normal tissues.  2) The release of immunosuppressive factors in the late stages of ferroptosis and ferroptosis of antitumor immune cells can negate the therapeutic effect.  3) OC cells can develop mechanisms to bypass ferroptosis: activation of alternative antioxidant mechanisms, restriction of PUFAs peroxidation, and the availability of labile iron.  4) Heterogeneity of OC and the absence of validated biomarkers that would help identify ovarian tumors prone to ferroptosis and select patients who would benefit from ferroptosis-suppressing therapy.  5) Lack of reliable methods for detecting and monitoring ferroptosis *in vivo*. |
| Ferroptosis | system Xc- | Erastin + cisplatin | HEY | **Preclinical trials**:  - *in vitro* cell culture - *in vivo* CDX | 132 |  |
| Ferroptosis | system Xc- | Erastin + cisplatin | A2780, A2780DDP (cisplatin-resistant) | **Preclinical trials**:  - *in vitro* cell culture | 133 |  |
| Ferroptosis | system Xc- ABCB1 | Erastin + docetaxel | A2780 (docetaxel-sensitive), A2780/Taxol (docetaxel-resistant) | **Preclinical trials**:  - *in vitro* cell culture | 134 |  |
| Ferroptosis | system Xc- SLC7A11 | Erastin + olaparib | *BRCA1/2* wild-type HEY, A2780 | **Preclinical trials**:  - *in vitro* cell culture - *in vivo* CDX | 135 |  |
| Ferroptosis | system Xc- LIP | Erastin Erastin + ferlixit | High LIP HEY, low LIP COV318 | **Preclinical trials**:  - *in vitro* cell culture - *in vivo* CDX | 136 |  |
| Ferroptosis | system Xc- GPX4 | Erastin + RSL3 | *KRAS*-mutant cells OVCAR-8, NCI/ADR-RES (doxorubicin-resistant OVCAR-8) | **Preclinical trials**:  - *in vitro* cell culture | 138 |  |
| Ferroptosis | Extracellular iron | ELU001 | Patients with advanced, recurrent cancers overexpressing folate-receptor alpha (N = 79), including OC | **Сlinical trials:** Phase I/II (safety, dosage, efficacy) The study was terminated early (lack of funds) | [NCT05001282](https://clinicaltrials.gov/study/NCT05001282) |  |
| Necroptosis | Caspases, IAPs, TNFR | TNF-α + IAP antagonists + zVAD | OVCAR-3, HEY; OV no. 2224 and OV no. 3971 cells from OC patients | **Preclinical trials**:  - *in vitro* cell culture | 161 | 1) In the advanced stage, OC may evade necroptosis (loss of RIPK3 and MLKL expression) or pyroptosis (GSDME suppression), which may lead to the inefficiency of some necroptosis- or pyroptosis-mediated therapies.  2) The dual pro-tumor and anti-tumor roles of necroptosis and pyroptosis: insufficient control or sustained induction may shift their role from activation of the immune system and immunogenic PCD to chronic tumor-supportive inflammation, which promoteы OC progression.  Consequently, the urgent task is to maximize the anti-tumor effect of necroptosis/pyroptosis while simultaneously reducing their pro-tumor effects.  3) Risk of developing significant off-target toxicity.  4) Like other non-apoptotic PCD, there is a lack of validated biomarkers and methods for detecting and monitoring pyroptosis/necroptosis *in vivo*. |
| Necroptosis | CD40, RIPK1/RIPK3/  MLKL axis | CD40L | LGSC-derived cells VOA1312 and MPSC1 | **Preclinical trials**:  - *in vitro* cell culture | 162 |  |
| Necroptosis | p62/RIPK1/RIPK3/MLKL axis | Sulfasalazine | SKOV3, SKOV3/DDP (cisplatin-resistant SKOV3) | **Preclinical trials**:  - *in vitro* cell culture | 163 |  |
| Necroptosis | ZBP1 => RIPK3/MLKL | Fisetin | OVCAR-3, A2780 | **Preclinical trials**:  - *in vitro* cell culture | 165 |  |
| Necroptosis | RIPK3/MLKL axis | Berberine + cisplatin | OVCAR-3 and 3 primary OC cells from patients with FIGO III serous OC | **Preclinical trials**:  - *in vitro* cell culture | 164 |  |
| Necroptosis | TNF-α/ RIPK1/RIPK3/ MLKL axis | Progesterone | *TP53* mutated PEO4, FE25 (fallopian tube fimbria epithelium, **origin of HGSOC**); *TP53*-/- mice (oviduct epithelium) | **Preclinical trials**:  - *in vitro* cell culture - *in vivo* mouse model | 166 |  |
| Pyroptosis (+ Apoptosis, Autophagy) | ROS (↑), MMP (↓) LC3-II (↑) GSDM-E | Osthole | OVCAR-3, A2780 | **Preclinical trials**:  - *in vitro* cell culture | 193 |  |
| Pyroptosis (+ Apoptosis, Autophagy) | ROS (↑), MMP (↓) LC3-II (↑) GSDM-E/-D | Nobiletin | OVCAR-3, A2780 | **Preclinical trials**:  - *in vitro* cell culture | 194 |  |
| Pyroptosis | Caspase-4, GSDME | Bexarotene | ES-2 | **Preclinical trials**:  - *in vitro* cell culture | 195 |  |
| Pyroptosis | Caspase-4,  GSDM-D | α-NETA | HO-8910 (ovarian serous adenocarcinoma), HO-8910PM (highly metastatic HO-8910), SKOV3, A2780 | **Preclinical trials**:  - *in vitro* cell culture - *in vivo* CDX (SKOV3) | 196 |  |
| Pyroptosis | Caspase-3,  GSDM-E | BI 2536 | A2780 | **Preclinical trials**:  - *in vitro* cell culture - *in vivo* CDX | 197 |  |
| Pyroptosis | Caspase-3,  GSDM-E | Paclitaxel | A2780 | **Preclinical trials**:  - *in vitro* cell culture | 198 |  |
| Pyroptosis | NLRP3 inflammasome | Tranilast | Cisplatin-resistant IGROV-CP20 | **Preclinical trials**:  - *in vitro* cell culture | 247 |  |
| Pyroptosis | SF3B1 => NLRP3/Caspase-3/  GSDM-E; PD-L1 | Pladienolide B Pladienolide B + αPDL1 | A2780, OVCAR-8; ID8 (mouse OSE cancer cells) | **Preclinical trials**:  - *in vitro* cell culture - *in vivo* CDX (ID8) | 249 |  |
